# Bioactive Compounds, Medicinal Benefits, and Contemporary Extraction Methods for Lemon Balm ( *Melissa officinalis* )

**DOI:** 10.1002/fsn3.70864

**Published:** 2025-09-07

**Authors:** Farhang Hameed Awlqadr, Ammar B. Altemimi, Syamand Ahmed Qadir, Othman Abdulrahman Mohammed, Mohammed N. Saeed, Mohammad Ali Hesarinejad, Naoufal Lakhssassi

**Affiliations:** ^1^ Food Science and Quality Control, Halabja Technical College Sulaimani Polytechnic University Sulaymaniyah Iraq; ^2^ Department of Food Science, College of Agriculture University of Basrah Basrah Iraq; ^3^ Medical Laboratory Techniques Department, Halabja Technical Institute, Research Center Sulaimani Polytechnic University Sulaymaniyah Iraq; ^4^ Medical Laboratory Science Department, Halabja Technical College Sulaimani Polytechnic University Sulaymaniyah Iraq; ^5^ Department of Nutritional Analysis and Health, Kifri Technical College Garmian Polytechnic University Kifri city Sulaimaniyah Province Iraq; ^6^ Department of Food Sensory and Cognitive Science Research Institute of Food Science and Technology (RIFST) Mashhad Iran; ^7^ Department of Biological Sciences, School of Science Hampton University Hampton Virginia USA

**Keywords:** antioxidant, bioactive compounds, extraction techniques, *M. officinalis*, pharmacological applications

## Abstract

Lemon balm ( *Melissa officinalis* ), a perennial herb belonging to the Lamiaceae family, is widely recognized for its medicinal properties and therapeutic benefits. This review offers a detailed exploration of the botanical features, phytochemical composition, and pharmacological uses of *M. officinalis* , highlighting key bioactive compounds such as phenolic acids (including rosmarinic and caffeic acids), flavonoids, essential oils (such as citral and citronellal), and triterpenoids (ursolic and oleanolic acids). Advanced extraction techniques, such as ultrasound‐assisted extraction (UAE), microwave‐assisted extraction (MAE), pressurized liquid extraction (PLE), supercritical fluid extraction (SFE), and matrix solid‐phase dispersion (MSPD), have greatly improved the efficiency of extraction, the preservation of bioactivity, and the sustainability of acquiring these bioactive compounds. Recent investigations have also shown its wide‐ranging pharmacological potential, which includes antioxidant, antimicrobial, anti‐inflammatory, neuroprotective, antiviral, antidepressant, anxiolytic, anticancer, cardioprotective, and cognitive‐enhancing properties. Furthermore, the traditional medicinal use of *M. officinalis* in managing neurodegenerative conditions such as stroke, epilepsy, dementia, and paralysis is increasingly supported by contemporary evidence, highlighting its therapeutic relevance in brain‐related disorders. However, further studies are necessary to refine extraction methods, standardize levels of bioactive compounds, and validate clinical applications. This review presents a critical synthesis of existing knowledge and future directions for integrating *M. officinalis* into nutraceutical, pharmaceutical, and cosmetic products.

## Graphical abstract

Lemon balm ( *Melissa officinalis* ) is a medicinal herb renowned for its diverse pharmacological properties, including antioxidant, antimicrobial, neuroprotective, and anti‐inflammatory effects. This review explores its botanical characteristics, bioactive compounds, advanced extraction methods, and therapeutic potential, particularly in treating brain‐related disorders. Future research is needed to optimize extraction processes and validate its clinical applications in nutraceutical, pharmaceutical, and cosmetic products.

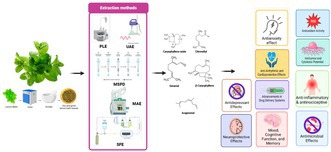

## 1 Introduction

Lemon balm ( *Melissa officinalis* ), a perennial herb belonging to the Lamiaceae family, has been known for its health properties and healing functions for a century. It is part of the Mediterranean and some parts of Asia, and it has gone through cultivation and subsequent uses in traditional formulations by the indigenous communities for hundreds of years (Ghazizadeh, Sadigh‐Eteghad, et al. 2021; Mathews et al. 2024). This plant is a natural remedy for anxiety, depression, digestive diseases, etc. Therefore, it has been extensively studied for its calming and mood‐enhancing properties. *M. officinalis* is a major medicinal herb, usually used as a potent anxiolytic, anticonvulsant, and sedative agent (Haybar et al. 2018). Recent scientific research has shown that aromatic plants and their biologically active molecules are among the many natural products that can contribute to the improvement of drugs or pharmacological activities (Christaki et al. 2012; Kieliszek et al. 2020; Qadir et al. 2025). *M. officinalis* contains diverse bioactive compounds, such as phenolic acids, flavonoids, rosmarinic acid (RA), and essential oils (EO) (Lešnik and Bren 2021; Khodja et al. 2018), which are associated with antioxidant, antimicrobial, anti‐inflammatory, neuroprotective, and antiviral properties (Virchea et al. 2021; Świąder et al. 2019). Consequently, it contributes to the prevention and supportive management of many different diseases, including infectious disorders and neurodegenerative diseases, through its bioactive effects on oxidative stress and inflammation (Doukani et al. 2021). The recent surge in research and innovation in the medical field has led to an increase in the production of natural products (Pham et al. 2019). *M. officinalis's* extraction of bioactive compounds has been the center of the latest research and development in traditional medicine. Methods such as maceration and steam distillation have been the only sources of EOs and other active components for decades (Žlabur et al. 2016). Several studies have indicated that the extraction method used significantly influences the yield and bioactivity of plant extracts. For instance, microwave‐assisted extraction (MAE) has been proven to enhance the recovery of phenolic compounds from *M. officinalis* compared with conventional methods (Golmakani and Rezaei 2008). Similarly, ultrasound‐assisted extraction (UAE) improved the antioxidant properties of *M. officinalis* extracts better by breaking down cell walls more effectively and allowing the solvent to get in better (Jovanović et al. 2022). Supercritical fluid extraction (SFE) using CO_2_ has also been shown to produce extracts with more volatile compounds and better antimicrobial properties (Reverchon and De Marco 2006). However, breakthroughs in extraction technologies, such as UAE, MAE, and SFE, have resulted in the enhancement of the efficiency and yield of bioactive compounds applied to them. These new methods ensure both the gentle treatment of plant material and the high quality of the resulting extracts (Garcia‐Vaquero et al. 2020; Wen et al. 2020). *M. officinalis* is useful in the pharmaceutical field (Dastmalchi et al. 2008; Scholey et al. 2014). Studies have provided evidence that its anxiolytic and antidepressant effects are the result of its ability to regulate different neurotransmitters such as gamma‐aminobutyric acid (GABA) and acetylcholine (Rodríguez‐Landa et al. 2022). Herpes simplex virus (HSV) activity has been effectively inhibited by the plant, which has validated its function in improving cognitive function (Chuanasa et al. 2008). Additionally, its antioxidative and anti‐inflammatory features that are essential for the cardiovascular system and the immune system make it a promising candidate for integrative medicine and functional foods (Aghababaei and Hadidi 2023). Although its use in therapeutic medicine is well documented, there is still a need for comprehensive research to fully delineate the mechanisms that underlie the medicinal properties of *M. officinalis* (Shakeri et al. 2016). Upcoming scientific work will concentrate on perfecting the extraction methods, standardizing the concentrations of bioactivities, and conducting clinical trials to demonstrate its efficiency among humans (Ghenabzia et al. 2023). Because of the growing focus on plant‐based therapies, a multidisciplinary approach is necessary to enable research teams to meet this demand and to provide better preconditions for applying *M. officinalis* in the new millennium healthcare system (Sharifi‐Rad et al. 2021). This review focuses on three main aspects of *M. officinalis* : its medicinal properties and bioactive compounds, including phenolic acids, flavonoids, RA, and EOs, along with their pharmacological activities; advances in extraction techniques, comparing traditional and modern methods and their impact on yield and bioactivity; and current and potential applications in therapeutic medicine, integrative health, and functional foods, with attention to future research needs.

## 2 History and Botanical Description

**FIGURE 1:**
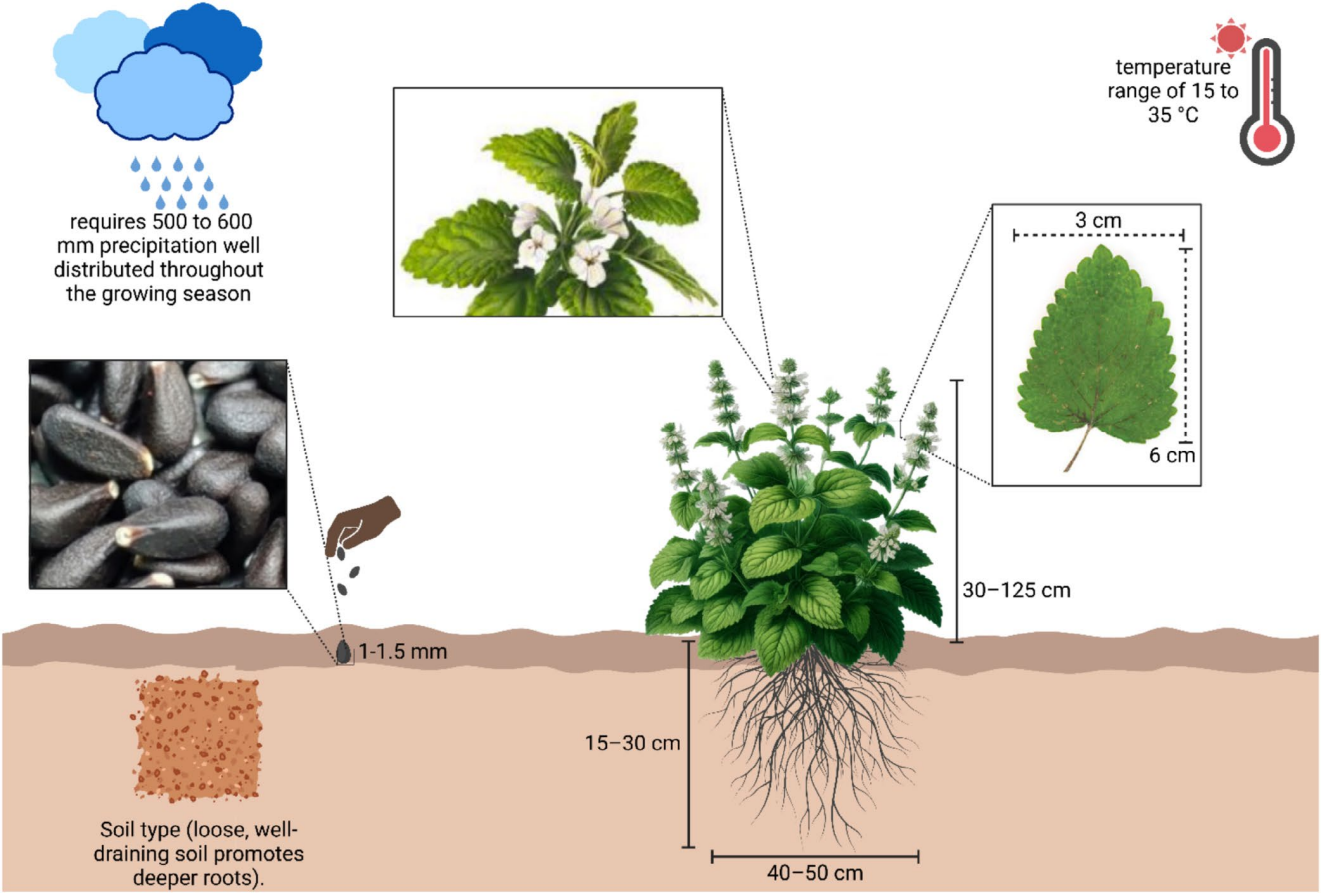
*M. officinalis* is a plant seen in the figure. Morphology, including seed shape (1–1.5 mm), leaf length (3 × 6 cm), and type of inflorescence, as well as a fibrous root system (15–30 cm deep, 40–50 cm lateral spread), is displayed in the drawing. The best soil for it is a loose, well‐drained one, with temperatures between 15°C and 35°C and a rainfall of 500–600 mm well distributed throughout the growth season. These conditions are the best for this type of plant. Created in BioRender. Awlqadr (2025e) https://BioRender.com/zpfkoj5.

*M. officinalis* is a perennial herb belonging to the Lamiaceae family. It has been grown for hundreds of years in the Mediterranean region, West Asia, and parts of Europe for its aromatic and medicinal uses. The meaning of “Melissa” comes from the Greek word for “honeybee,” which means the use of this plant in the past to attract bees that produce high‐quality honey (Basar and Zaman 2013; Ullah and Hassan 2022). *M. officinalis* has been used for its calming ability in the digestive tract and as a remedy for wounds for a long time in both the Greek and Roman eras. In ancient history, there came a bright moment for *M. officinalis* as it became a part of Saracen medicine. It was, among other things, used in the treatment of insomnia, as well as panic attacks, palpitations, and indigestion associated with overthinking (Abdel‐Naime et al. 2016; Ghazizadeh, Mohammadinasab, et al. 2021). *M. officinalis* has been commonly used historically for soothing the digestive tract, easing anxiety, and helping sleep. Recent scientific studies have confirmed some of these effects: *M. officinalis* extracts have been reported to have anxiolytic, sedative, and cognition‐enhancing effects, with tangible improvements in mood and memory performance in healthy subjects (Kennedy et al. 2002), and also exert antioxidant, anti‐inflammatory, and antimicrobial effects through bioactive compounds such as RA and flavonoids (Świąder et al. 2019). These findings justify its traditional use as a medicinal agent and signify its potential in contemporary phytomedicine. *M. officinalis* is a bushy herbaceous plant that attains a height of 30–125 cm, characterized by soft, short hairs covering all its parts. The stem is upright, branching, often smooth, and quadrangular. Leaves are petiolate and ovate, measuring up to 6 cm in length and 3 cm in width, with a cuneate top surface and a cordate base, exhibiting crenate teeth, subglabrous texture, and occasionally featuring glandular hairs or punctate glands on the underside (Komarov et al. 1977; Abdel‐Naime et al. 2020). The leaves emit a lemon‐like aroma and flavor owing to the presence of volatile chemicals, specifically monoterpenes and sesquiterpenes (Aharizad et al. 2012; Oladeji et al. 2019; Hetka et al. 2018). Flowers are white or pale pink, including tiny clusters of 4–12 flowers over the summer. It possesses two stamens and 4‐lobed ovaries, resulting in the formation of one to four nutlets. The seeds are approximately 1–1.5 mm long and have an ovate shape with a dark brown or black hue. *M. officinalis* thrives at temperatures between 15°C and 35°C and requires 500–600 mm of evenly distributed precipitation during the growing season; otherwise, irrigation is required (Saeb and Gholamrezaee 2012; Waheed et al. 2020). *M. officinalis* possesses a fibrous root structure characterized by numerous lateral roots, enhancing the plant's adaptability to varying environmental circumstances. The aerial portions of the plant perish with the onset of winter, while fresh shoots arise from the roots at the beginning of spring (Turhan 2006; Yakimova and Yegorova 2021). The main features of the morphology and conditions of *M. officinalis* and the list of the best scents are shown in Figure 1. The taxonomical classification of this plant is as follows: kingdom: Plantae/Subkingdom: Tracheobionta/Super‐division: Spermatophyta/Division: Magnoliaophyta/Class: Magnoliopsida/Sub‐class: Asteridae/Order: Lamiales/Family: *Lamiaceae*/Genus: *Melissa L*./Species: *officinalis L*. (Basar and Zaman 2013).

## 3 Methodology and Literature Search Strategy

A comprehensive literature search was conducted between 2000 and 2025 in PubMed, Scopus, Web of Science, and Google Scholar to identify relevant studies on *M. officinalis* . Search terms included combinations of “ *Melissa officinalis* ” OR “lemon balm” with “bioactive compounds,” “phytochemicals,” “extraction,” “UAE,” “MAE,” “SFE,” “PLE,” “MSPD,” “pharmacological activities,” and “therapeutic applications.” The inclusion criteria comprised English‐language articles (2000–2024) reporting experimental, analytical, or clinical data on the plant's composition, extraction methods, and pharmacological effects; key earlier works were also considered. Non‐English papers without translations, non‐scientific reports, and studies unrelated to *M. officinalis* were excluded. The reference lists of included papers were screened for additional sources.

## 4 Phytochemical Composition of *M. officinalis*

*M. officinalis* is a well‐documented medicinal herb with a complex and diverse phytochemical composition that contributes to its pharmacological properties (Riaz et al. 2016). The chemical composition analysis of *M. officinalis* leaves, as presented in Table 1, indicated a diverse nutritional and phytochemical profile. The moisture content was moderate (8.99%), suggesting effective drying processes that facilitate preservation. Notably, *M. officinalis* leaves exhibited a relatively high protein content (13.50%), highlighting their potential as a nutrient‐rich additive in food formulations. The cellulose content, recorded at 26.56%, underscores the significant dietary fiber presence beneficial for digestive health. Moreover, the phytochemical analysis reveals substantial amounts of chlorophyll, particularly chlorophyll b at 36.82 μg g^−1^ dry weight, suggesting strong antioxidant potential. This is further supported by the presence of significant phenolic (184.33 mg GAE g^−1^) and flavonoid (12.65 mg QE g^−1^) contents, emphasizing the functional potential of *M. officinalis* leaves as bioactive ingredients in nutraceutical and pharmaceutical applications. The low EO yield (0.03% v/w) is characteristic but should be noted for consideration in EO extraction processes and applications (Doğan et al. 2021). The plant has been traditionally used in various medicinal systems, and modern phytochemical investigations have identified numerous bioactive compounds responsible for its therapeutic effects. Among its most important constituents, the EOs present in *M. officinalis* have been extensively studied. These oils are rich in monoterpenes and sesquiterpenes, including citronellal, geranial, neral, and limonene, which contribute to their distinct lemon‐like aroma (Petrisor et al. 2022; Moradkhani et al. 2010). The EO composition extracted from dried *M. officinalis* leaves, analyzed separately (Table 2), exhibited a diverse array of major bioactive constituents, including geranial (citral A), neral (citral B), caryophyllene oxide, β‐caryophyllene, and citronellal, highlighting the pharmacological and aromatic importance of this plant. Minor compounds, though present in lower percentages, collectively contribute to the EO's overall therapeutic potential and fragrance profile (Sharafzadeh et al. 2011). The fatty acid profile of *M. officinalis* leaves varied by geographic origin (Table 3). These bioactive constituents were obtained using solvent extraction followed by gas chromatography (GC) analysis, a standard method for lipid profiling in plant tissues. Linoleic acid (C18:2 n‐6) was predominant, ranging from 66.74% (Nefza) to 74.08% (Germany). Palmitic (C16:0) and oleic (C18:1 n‐9) acids were also abundant. Notably, palmitoleic acid (C16:1) was absent only in the French sample, reflecting the influence of environmental conditions on composition and potential applications (Hong et al. 1996). Other compounds such as geraniol, thymol, and linalool are rich in oxygen and strengthen their anti‐inflammatory and antimicrobial activities (Hăncianu et al. 2008). The blend of the oil can change subject to both the environmental factors and the geographical position of the plant, but research showed its high‐level protection from bacteria and fungi, mainly the bacteria that are familiar to the common population (Brochot et al. 2017; Jalal et al. 2015). *M. officinalis* is a very rich source of the compound palmitic acid and has a high content of polyphenolic compounds, including phenolic acids and flavonoids (Petrisor et al. 2022). The most representative phenolic acid in *M. officinalis* is RA, which has been acknowledged globally as one of the greatest antioxidants ever. It also has a wide spectrum of neuroprotective and anti‐inflammatory qualities (Barros et al. 2013). Besides RA, other relevant phenolic acids that are found in *M. officinalis* are chlorogenic acid, caffeic acid, and ferulic acid, which all contribute to their cytoprotective and radical scavenging effects. In addition to luteolin, quercetin, rhamnetin, and apigenin, flavonoids are also present in a large amount, which makes possible the enhancement of the pharmacological potential of the plant. Some of these compounds interact with inflammatory markers and help promote cognitive function, which is why *M. officinalis* can be used in protective and mood‐enhancing applications (Petrisor et al. 2022). One of the main bioactive compounds identified in the species is terpenoids, particularly triterpenes such as ursolic acid and oleanolic acid (Petrisor et al. 2022). These compounds have been proven to have notable anti‐inflammatory and anticancer properties, and they may be used successfully as complementary therapies for several chronic diseases (Kashyap et al. 2016). The research studies convey that the extracts from *M. officinalis* can block the proliferation of cancer cells and provoke apoptosis in some cancer cell lines, affirming, meanwhile, that these triterpenoids may have a leading role in their therapeutic effects. In addition, there are other, although less commonly known, yet biologically important compounds, such as aldehydes, glycosides, and tannins, which are also contained in the plant, thus increasing its medicinal worth. Tannins, among others, are polyphenol molecules that confer plant astringency and may help with heart health by modulating oxidative stress and inflammation (Faraji et al. 2021). The chemical profile of *M. officinalis* may have changed according to different environmental factors, cultivation methods and differences in the extraction procedures (Table 4). The initial harvest's analysis of *M. officinalis* leaves showed the mineral composition differences between normal full irrigation (CFI) and organic full irrigation (OFI). *M. officinalis* grown under OFI showed even higher levels of phosphorus (P), magnesium (Mg), zinc (Zn), and copper (Cu) than the plants grown under CFI, implying that the organic culture of the plant alone might be sufficient as a nutrient supplier and have benefits for the health of the plant (Chrysargyris et al. 2022). Recent investigations have looked into the best extraction method, where bioactive compounds obtain full therapeutic potential (Uwineza and Waśkiewicz 2020). The studies have established that SFE techniques using ethanol‐based extractions render the highest amounts of RA and other phenolic compounds. However, widely proposed alternative extraction methods, such as ultrasound‐assisted and SFE, have been analyzed for their effects on the yield of EOs and other phytochemicals (Rodríguez‐Solana et al. 2015; Osorio‐Tobón 2020). The differences in the phytochemical composition of the different types of species each group of individuals grows in different regions mean that research must make every effort to harmonize production (Castro‐Muñoz et al. 2023). Researchers have isolated and characterized major phytochemicals from various parts of *M. officinalis*, including the less‐studied root and stem tissues. Table 5 summarizes the key chemical constituents identified in these parts, along with their classifications and corresponding references. Altogether, *M. officinalis is* a multifaceted herbal medicine with a wide range of bioactive elements, including EOs, phenolic acids, flavonoids, terpenoids, and tannins, each responsible for a broad spectrum of pharmacological activities. Its antioxidant, antimicrobial, anti‐inflammatory, neuroprotective, and potential anticancer properties make it a valuable herb in traditional and modern medicine. The more studies on the plant's phytochemical composition, mechanisms of action, and medicinal applications, the better it will be defined as the main solution in alternative medicine (Shakeri et al. 2016).

**TABLE 1:** Chemical characterization of *M. officinalis* leaves (Doğan et al. 2021).

Parameters	Concentration
Moisture (%)	9
Essential oil yield (% v/w)	0.03
Protein content (%)	13.5
Cellulose content (%)	26.56
Ash content (%)	9.9
Chlorophyll (a)	32
Chlorophyll (b)	37
Total carotenoids	1.90
Total flavonoid content	12.65
Total phenolic content	184.3

**TABLE 2:** Range of natural production (%) of the oil abstracted from *M. officinalis* (Petrisor et al. 2022; Nouri et al. 2020; Nurzyńska‐Wierdak et al. 2014).

Compound name	Concentration (%)
Major Constituents (> 5%)	
Caryophyllene oxide	1.3–44
(E)‐ Caryophyllene	1–6.8
Geranial	6.2–51
Citronellal	0.4–20
Neral (citral B)	4.3–35
Geranyl acetate	0.5–19
α‐Copaene	0.1–7.02
α‐Cadinol	5.6
β‐Caryophyllene	1.3–29.14
Minor Constituents (< 5%)	
(E)‐Nerolidol	0.2
(2E)‐Nonen1al	0.2
(E)‐β‐Ionone	0.9
(E–E)‐Geranyl linalool	1.59
(E) α‐Bergamotene	1.24
(E)β‐Ocimene	0.1–0.5
1,2‐Benzenedicarboxylic acid ester	0.6
(Z)‐β‐Ocimene	0.1
14‐Hydroxy‐9‐epi‐(E) Caryophyllene	0.2
1,8‐Dehydrocineol	0.1
3,5‐Heptadienal,2‐ethylidene‐6‐methyl	0.4
1‐Octen‐3‐ol	0.2–0.3
6‐Methyl‐5‐hepten‐2‐ol	0.2–1.7
3‐Octanone	0.2
Benzene acetaldehyde	0.3
Camphor	0.1–0.4
Camphene	0.38–1.38
Isogeranial	1.4–2.0
Isomenthol	2.4
Iso Aromadendren epoxide	0.46
Germacrene D	0.2–2.0
Di‐hydrocitronellol acetate	0.3
Humulene epoxide II	0.2–1.29
Cis‐chrysanthenol	0.7–1.70
Geraniol	0.6–0.7
Citronellol	0.4–1.89
Cis‐Rose oxide	0.1–0.2
Citronellyl acetate	0.1
Cis‐2H‐3a‐Methyl‐octahydro‐Inden‐2‐one	4.7
Carvaceol	0.3–1
Caryophyllenol	0.5–2.23
β‐Pinene	1.1
Linalool + trans‐Sabinene hydrate	0.5–0.8
Thymol	0.1–3.1
β‐sesqui‐phellandrene	0.97
γ‐Cadinene	0.76–1.77
Sabinene	0.4
Methyl citronellate	0.5–2.78
α‐Humulene	0.2–2.6
n‐Heneicosane	0.4
n‐Nonanal	0.1–0.4
Phytol	3.64
α‐Calacorene	0.76
Myrcene	0.1–0.3
Nerol	0.2
trans‐Limonene oxide	0.6
p‐Cymene	0.1
Menthol	0.3
α‐Cubebene	0.42–1.23
Methyl eugenol	0.1
Rosefuran epoxide	0.6–0.7
Linalool	0.3–0.5
para‐Mentha‐1 (7), 8‐diene	0.1
γ‐Terpinene	0.3–0.5
Neryl acetate	0.1
t‐Muurolol	0.59
Methyl geranate	0.2–0.4
β‐Cubebene	0.1
n‐Eicosane	0.6

**TABLE 3:** *M. officinalis* leaves showing the variations in the percentage of fatty acids composition from the different places where they are grown (Souihi et al. 2020).

Fatty acid	France (%)	Germany (%)	Tabarka (%)	Nefza (%)
Linoleic acid	73.9	74	71	67
Arachidic acid	1.6	1	1.1	1.3
Palmitic acid	16.3	15.8	15.8	13.3
Oleic acid	4.62	6.29	5.89	4.26
Palmitoleic acid	—	1.71	1.00	1.47
Total (%)	95.38	98.91	94.65	88.02

**TABLE 4:** Mineral composition of *M. officinalis* leaves under conventional full irrigation (CFI) and organic full irrigation (OFI) during the first harvest (Chrysargyris et al. 2022).

Mineral	CFI	OFI
N (g/kg)	19.89 ± 0.22	18.26 ± 0.63
K (g/kg)	32.74 ± 0.41	30.07 ± 0.14
P (g/kg)	2.49 ± 0.03	3.35 ± 0.13
Ca (g/kg)	23.42 ± 1.33	20.01 ± 1.88
Mg (g/kg)	0.38 ± 0.03	0.43 ± 0.01
Na (g/kg)	0.35 ± 0.01	0.28 ± 0.01
Zn (mg/kg)	28.83 ± 0.82	49.96 ± 3.08
Cu (mg/kg)	125.89 ± 20.46	266.43 ± 44.50

*Note:* Values represent mean ± SD (*n* = 3).

**TABLE 5:** Major phytochemicals isolated and characterized from *M. officinalis* (root and stem).

No.	Classification	Chemical component	Part of plant	References
1	Phenolic acids	Rosmarinic acid	Root	Mimica‐Dukic et al. (2004)
2	Triterpenoids	Ursolic acid	Stem	Yahyazadeh et al. (2017)
3	Flavonoids	Luteolin	Root	Wee et al. (2019)
4	Phenolic acids	Caffeic acid	Root	Zgórka and Głowniak (2001)
5	Triterpenoids	Oleanolic acid	Root	Yahyazadeh et al. (2017)
6	Lignans	Pinoresinol	Stem	Zhao et al. (2023)
7	Volatile compounds	β‐Caryophyllene	Stem	Cho et al. (2003)
8	Coumarins	Scopoletin	Root	Wee et al. (2019)
9	Alkaloids (minor)	Actinidine (trace amounts)	Root	Kim et al. (2006)
10	Flavonoids	Apigenin	Stem	Sentkowska et al. (2015)

## 5 Methods of Extraction for Compounds From *M. officinalis*

### 5.1 Solvent Extraction

**FIGURE 2:**
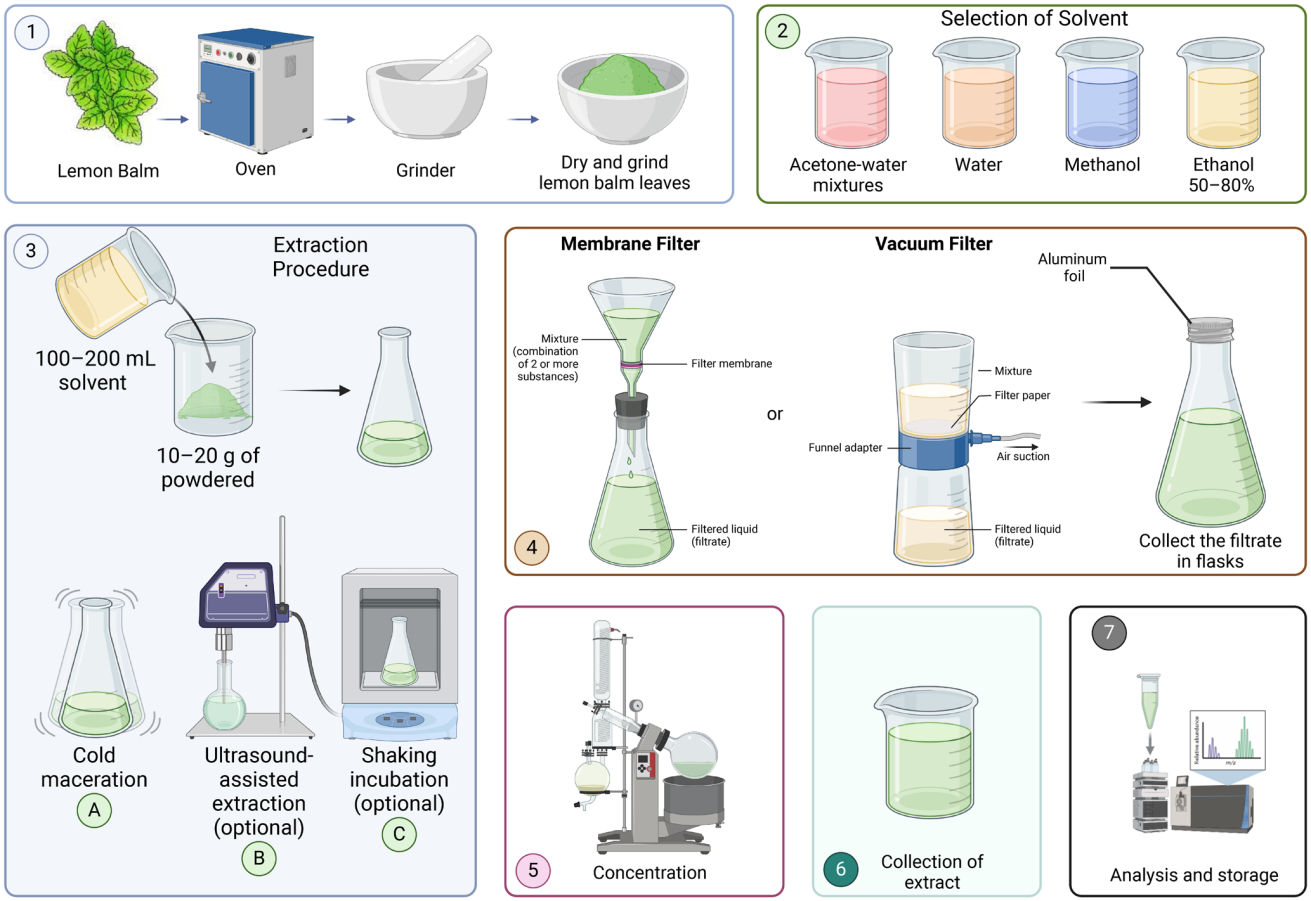
The figure shows sample preparation (drying and grinding), solvent selection (acetone‐water, water, methanol, and 50%–80% ethanol), extraction methods (cold maceration, ultrasound‐assisted, and shaking incubation), membrane and vacuum filtration, extract concentration, and extract collection, analysis, and storage. Created in BioRender. Awlqadr (2025f) https://BioRender.com/hz5j22e.

Ethanol and hydro‐alcoholic extractions are highly effective methods for isolating bioactive compounds, including phenolic acids such as RA, from *M. officinalis* . As illustrated in Figure 2, hydro‐alcoholic solutions, particularly 70% ethanol, provide optimal polarity to extract bioactive compounds like RA, ursolic acid, and oleanolic acid, known for their potent antioxidant activities. The extraction procedure typically involves drying, grinding the plant material, and using selected solvents such as ethanol‐water mixtures (Figure 1, steps 1 and 2). Enhanced extraction efficiency is achieved at elevated temperatures (50°C–65°C), low liquid‐to‐solid ratios (e.g., 5:1), and moderate extraction times (30 min–2 h), which facilitate the release of bioactive compounds from plant matrices through methods like cold maceration, UAE, or shaking incubation (Figure 1, step 3; Peev et al. 2011). Filtration, concentration, and subsequent collection and analysis steps (Figure 1, steps 4–7) ensure compound stability and high yields, making ethanol‐based extraction scalable and suitable for industrial applications (Herodež et al. 2003). Comparative studies have demonstrated that ethanol extraction outperforms alternative solvents like deep eutectic solvents in preserving bioactivity, including antioxidant and antimicrobial properties (Dheyab et al. 2021; Socas‐Rodríguez et al. 2021). Thus, as outlined in Figure 1, ethanol‐based extraction is crucial for efficiently and sustainably extracting phenolic compounds, supporting their applications in nutraceuticals, cosmetics, and pharmaceuticals (Delueg et al. 2019). Further optimization of ethanol concentration and extraction parameters can enhance efficiency and industrial scalability. Among the various extraction solvents, water is considered the safest and most environmentally friendly, though it may extract fewer non‐polar compounds. Methanol and ethanol are highly efficient for extracting phenolic compounds, with ethanol preferred for its lower toxicity and food‐grade status. Acetone‐water mixtures often enhance polyphenol extraction due to their intermediate polarity and ability to disrupt plant cell walls more effectively. While methanol provides strong extractive power, especially for low molecular weight polyphenols, its toxicity limits its application in consumables. Ethanol balances safety and efficacy, making it ideal for food and pharmaceutical applications (Ali Redha 2021; Kajdzanoska et al. 2011).

### 5.2 UAE

**FIGURE 3:**
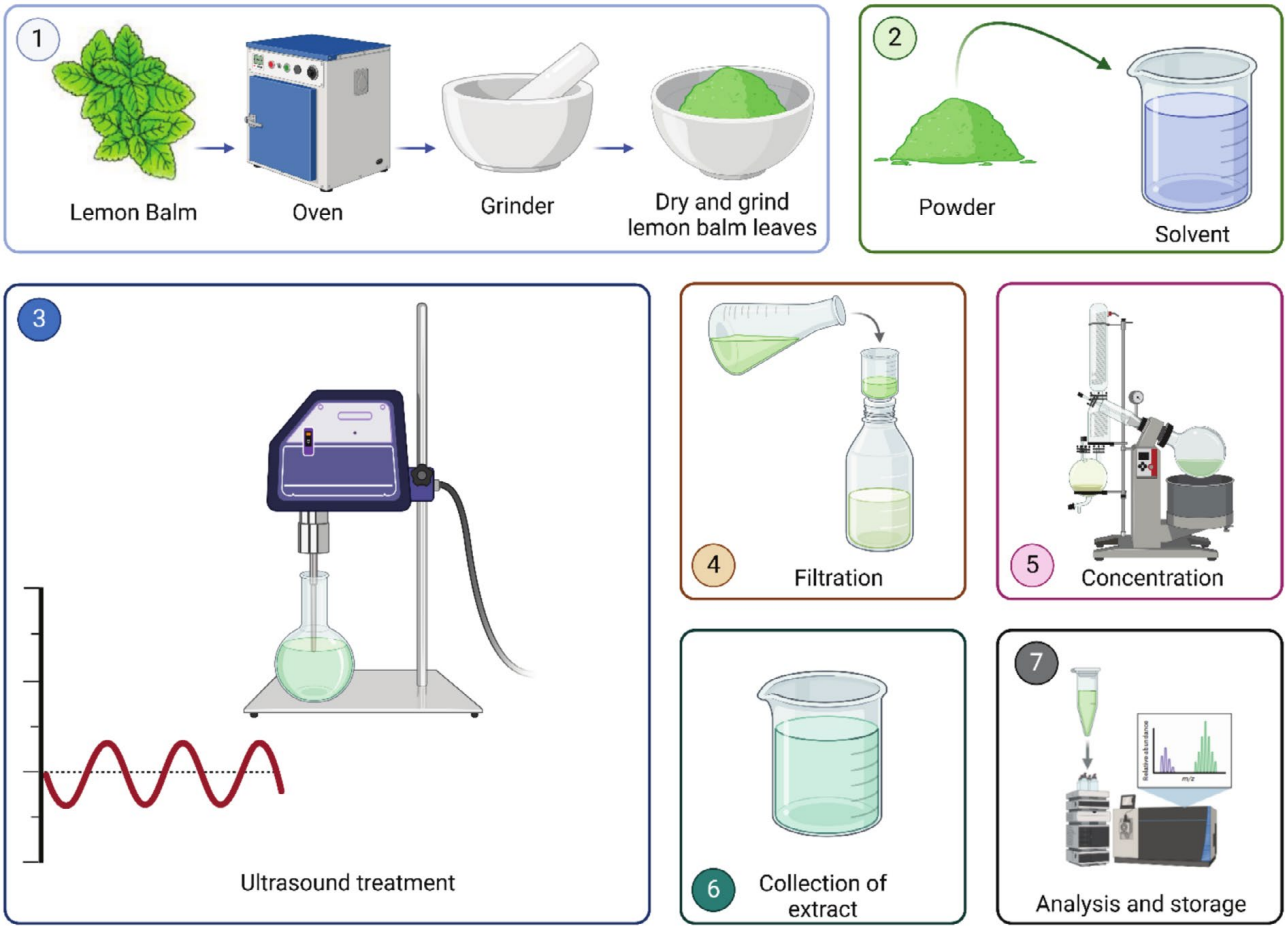
Schematic representation of the ultrasound‐assisted extraction (UAE) procedure for bioactive compounds from *M. officinalis* leaves. The process includes drying and grinding of plant material, ultrasonic extraction, filtration, concentration, and subsequent analysis and storage of extracts. Created in BioRender. Awlqadr (2025a) https://BioRender.com/oyk8r1m.

UAE is a highly efficient and eco‐friendly technique for extracting bioactive compounds from *M. officinalis*, as illustrated in Figure 3. This method utilizes ultrasonic vibrations to disrupt plant cell walls, enhancing the release of valuable phytochemicals. Optimized UAE conditions, including an ultrasonic power of 371.7 W, an extraction time of 33 min, and a solvent ratio of 40% ethanol‐water, result in notably high yields of RA, reaching up to 86.3 mg/g of dry weight, exceeding conventional heat‐based and microwave‐assisted methods (Caleja, Ribeiro, et al. 2017; Carreira‐Casais et al. 2021). Furthermore, UAE extracts demonstrate superior antioxidant activity, attributed to better preservation of heat‐sensitive phenolic compounds, such as flavonoids and RA, critical for their potent free‐radical scavenging capabilities (Saifullah et al. 2020; Chung et al. 2010). For example, Dahmoune et al. (2015) demonstrated that UAE of *Myrtus communis* leaves produced extracts with greater phenolic content and antioxidant activity than Soxhlet or infusion methods. These findings highlight UAE as a practical and efficient technique for recovering bioactive antioxidants while minimizing thermal degradation (Dahmoune et al. 2015). Comparative analyses indicate that UAE significantly reduces extraction times and thermal degradation of compounds, emphasizing its advantage over traditional extraction techniques (Jovanović et al. 2023). Dahmoune et al. (2014) found that UAE of *Myrtus communis* leaves led to higher recovery of total phenolics and reduced solvent and energy consumption, underlining the eco‐efficiency of the technique. These findings confirm that UAE not only improves extraction kinetics but also enhances the quality and bioactivity of phytochemical extracts (Dahmoune et al. 2015). UAE also aligns with sustainable extraction principles, minimizing solvent and energy use while supporting diverse applications in nutraceuticals, pharmaceuticals, and cosmetics (Chemat et al. 2017; Khalid et al. 2024; Shen et al. 2023). It presents challenges when extracting sensitive bioactives from *M. officinalis* . Key compounds such as RA and flavonoids may degrade due to localized heating or radical formation under high ultrasonic intensity or prolonged exposure (Chemat et al. 2017). UAE can also lead to emulsification and complicate purification steps. These limitations can be mitigated by optimizing ultrasound parameters (e.g., using pulsed instead of continuous waves), operating at lower temperatures, and selecting appropriate solvent systems. Combining UAE with techniques like MAE or enzymatic pretreatment has also been shown to improve efficiency and compound stability (Dahmoune et al. 2015). Thus, while UAE is promising, careful control of process conditions is essential for preserving the integrity of *M. officinalis* phytoconstituents.

### 5.3 Pressurized Liquid Extraction

**FIGURE 4:**
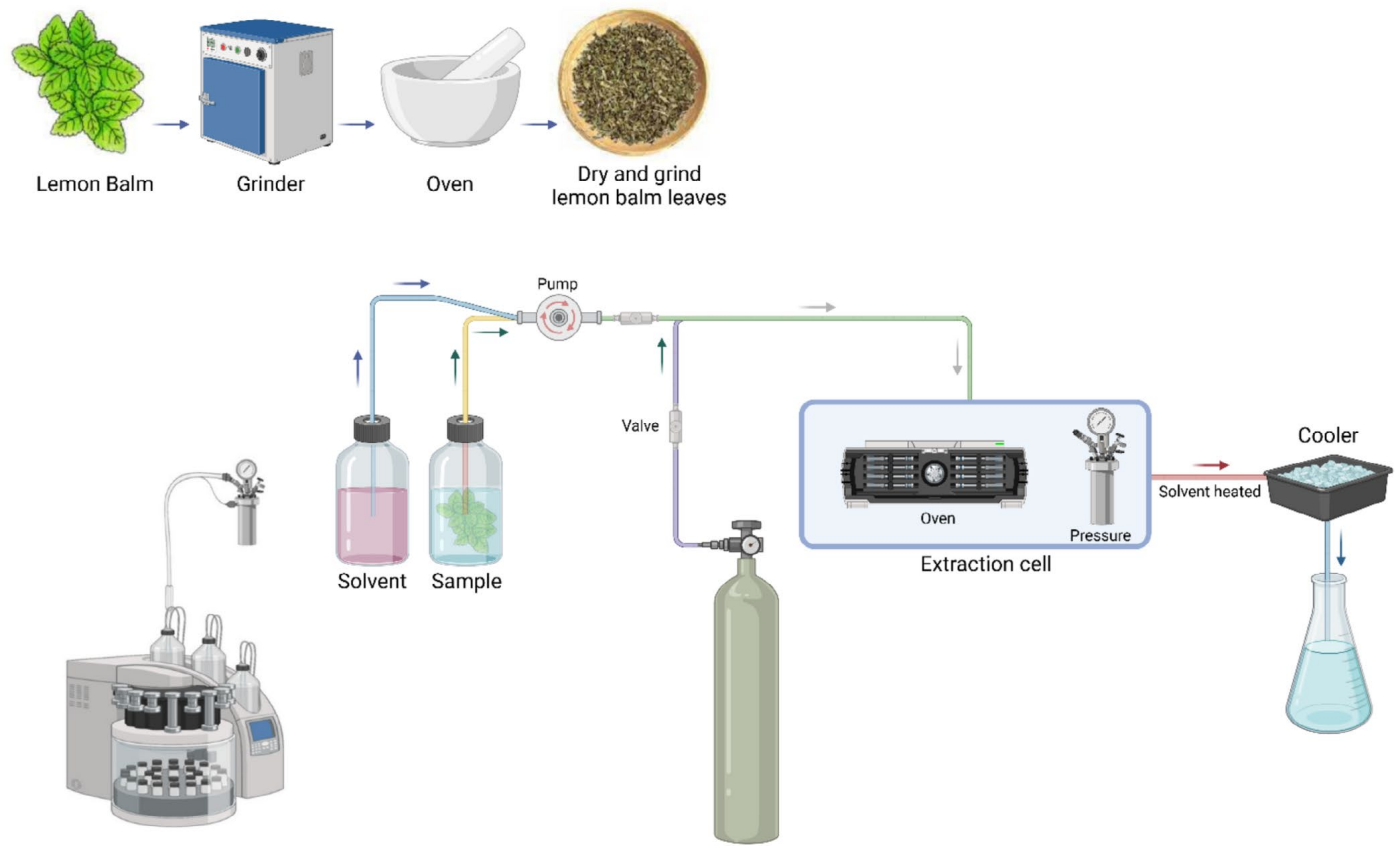
Pressurized liquid extraction (PLE) schematic for *M. officinalis* leaf bioactive chemicals. Examples of sample preparation, solvent administration, pressured extraction in a heated extraction cell, cooling, and final extract collection for analysis. Created in BioRender. Awlqadr (2025b) https://BioRender.com/it63156.

Pressurized liquid extraction (PLE), also known as accelerated solvent extraction (ASE), has emerged as an efficient technique for extracting bioactive compounds such as phenolic acids and flavonoids from *M. officinalis*, as illustrated in Figure 4. This method involves extraction under elevated pressure and temperature, keeping solvents like ethanol and water in their liquid state above their normal boiling points, significantly improving solubility, diffusion rates, and extraction efficiency. Optimized PLE conditions, particularly temperatures around 150°C combined with 40%–70% ethanol concentrations, have been shown to enhance yields of RA and other phenolics compared to conventional extraction methods such as Soxhlet or enzyme‐assisted extraction. For example, water‐based PLE can achieve phenolic contents as high as 193.18 mg GAE/g of extract, with preserved antioxidant activities confirmed through DPPH and TEAC assays (Miron et al. 2013). In addition to improved yields, PLE offers significant sustainability advantages, reducing solvent usage, extraction time, and energy consumption, which makes it suitable for commercial‐scale production of nutraceuticals, pharmaceuticals, and cosmetic ingredients while preserving the biological activity of sensitive compounds (Raut et al. 2015). In contrast, although UAE is also considered a green extraction method, it presents certain limitations when applied to *M. officinalis* . High‐intensity ultrasound or prolonged sonication can lead to localized overheating and the formation of reactive oxygen species, which may degrade thermolabile phytoconstituents such as RA, luteolin, and caffeic acid derivatives. Furthermore, UAE can cause foaming or emulsification, which complicates downstream purification processes and affects extract consistency. These drawbacks may compromise both yield and bioactivity unless carefully mitigated by optimizing operational parameters such as pulse duration, solvent selection, temperature control, and extraction time. In this context, PLE/ASE has been demonstrated as a more stable and scalable alternative. For instance, Miron et al. (2013) employed ASE to successfully extract high yields of RA and total phenolics from *M. officinalis* , demonstrating higher extraction efficiency and better preservation of antioxidant activity compared to conventional methods. Also, Miron et al. (2011) showed that PLE using ethanol–water mixtures at moderate temperatures (200°C) significantly improved flavonoid recovery from *M. officinalis* without degrading sensitive compounds. These studies emphasize that while UAE is effective for small‐scale and rapid extractions, PLE is more robust and consistent for industrial applications requiring high‐quality botanical extracts (Miron et al. 2011).

### 5.4 SFE

**FIGURE 5:**
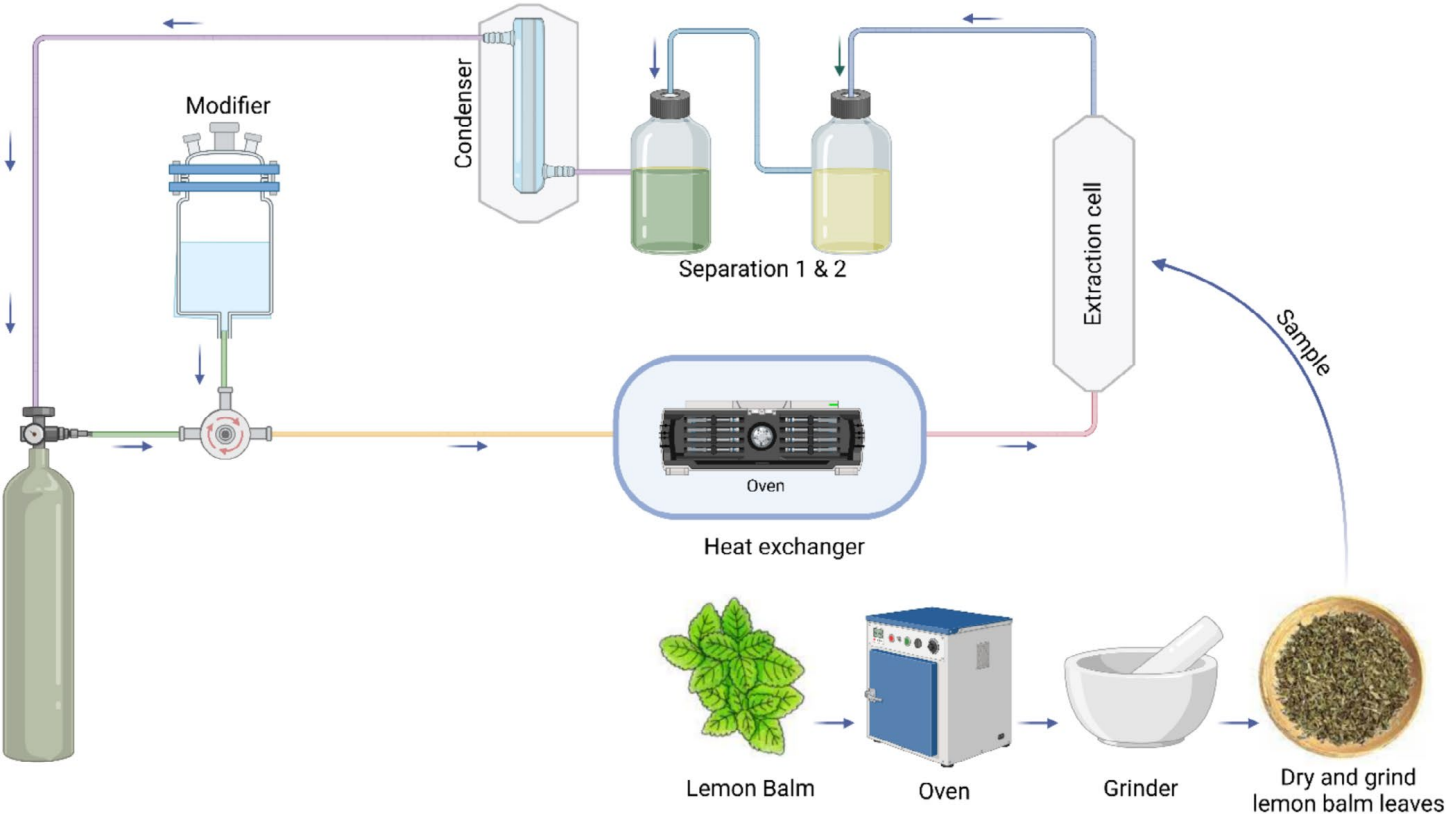
Schematic representation of the supercritical fluid extraction (SFE) process utilizing supercritical CO_2_ for extracting bioactive compounds from *M. officinalis* leaves. The figure illustrates sample preparation, extraction under supercritical conditions, extract separation and collection, and solvent recycling. Created in BioRender. Awlqadr (2025c) https://BioRender.com/bbqaegb.

SFE, depicted in Figure 5, is a green, efficient, and innovative technique used to extract bioactive compounds, including volatile oils (citral, citronellal, and geraniol) and phenolic acids (RA, caffeic acid, and chlorogenic acid), from *M. officinalis* . Employing supercritical carbon dioxide (CO_2_) as a solvent, SFE operates under carefully controlled pressures (typically 10–18 MPa) and moderate temperatures (around 308–313 K). These conditions significantly enhance compound solubility, diffusion, and extraction selectivity without causing thermal degradation. Previous studies indicate that SFE effectively isolates valuable EOs, such as citral and citronellal, and polyphenols with potent antioxidant properties validated by assays like Rancimat and spectrophotometric analyses. For example, optimized extraction conditions at 10 MPa and 323 K over 30‐min yield exceptionally high levels of phenolic compounds (Raut et al. 2015; Mustafa and Turner 2011). This extraction technique aligns with sustainable chemistry principles by eliminating toxic organic solvents, making SFE ideal for generating high‐purity extracts suitable for pharmaceutical, nutraceutical, and cosmetic formulations. Extraction of *M. officinalis* using the SFE is fast, efficient, and ecologically friendly; however, its drawbacks include relatively poor extraction of polar phenolic compounds, as CO_2_ is a non‐polar solvent, leading to lower antioxidant activity compared to solvent‐borne extracts. SFE was found to be less effective and relatively sensitive to pressure and temperature regarding phenolic content and antioxidant power compared to ethanol–water extraction (García‐Risco et al. 2017). These drawbacks can be overcome with the use of co‐solvents or by combining SFE with other methods such as UAE (Villalva et al. 2021).

### 5.5 MAE

**FIGURE 6:**
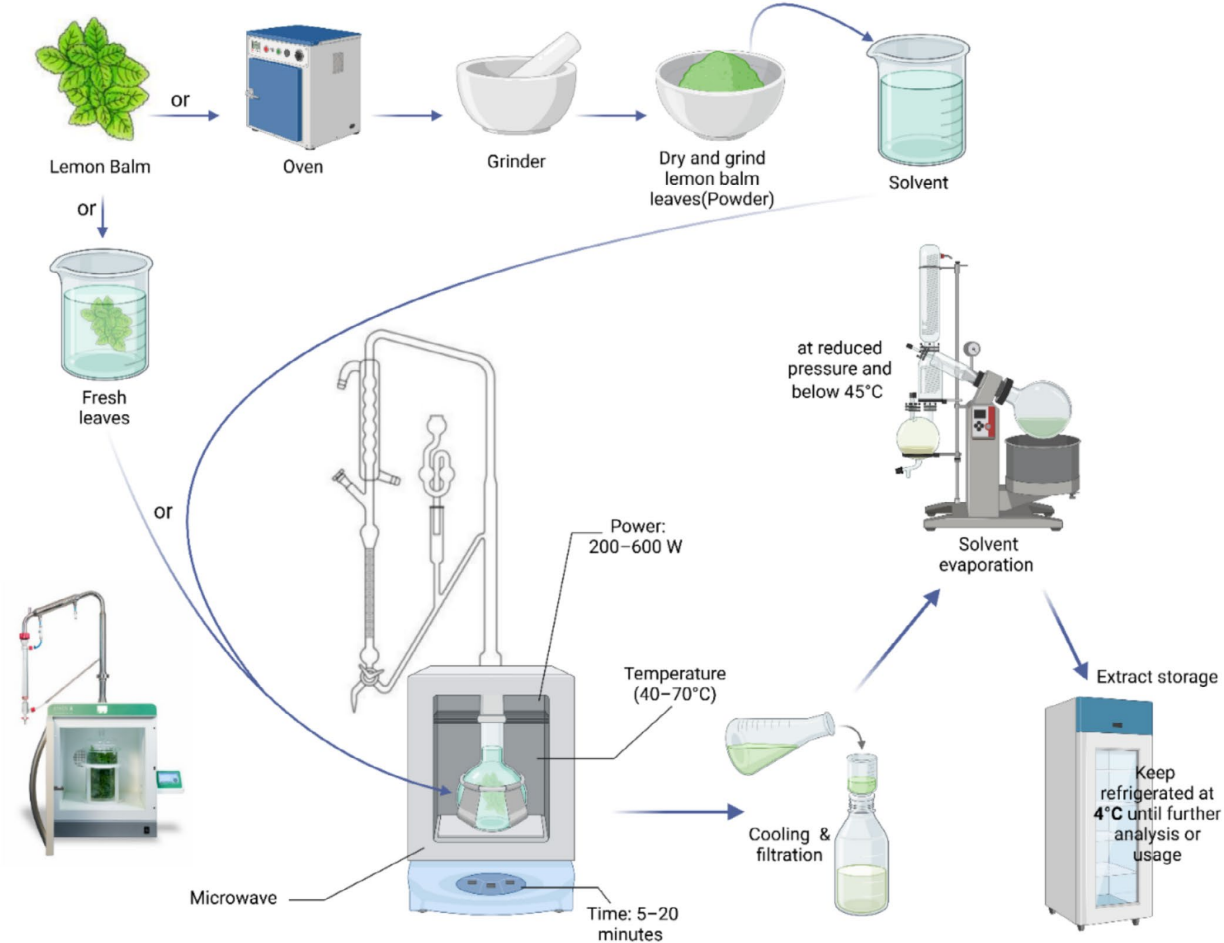
Bioactive chemical extraction from *M. officinalis* leaves using microwave‐assisted extraction (MAE). The graphic shows sample preparation (dry or fresh leaves), solvent selection, microwave extraction, chilling, filtering, solvent evaporation, and extract storage. Created in BioRender. Awlqadr (2025d) https://BioRender.com/gr3vvm1.

MAE, illustrated in Figure 6, is an innovative and highly efficient technique for extracting bioactive compounds such as phenolic acids (RA, caffeic acid) and flavonoids (luteolin, apigenin) from *M. officinalis* . The MAE method utilizes microwave radiation to rapidly heat both plant material and solvent, significantly enhancing solvent penetration and accelerating the extraction of valuable compounds. Optimal conditions—such as using hydro‐alcoholic solvents (50%–70% ethanol), applying microwave power around 371.7 W, and short extraction times of 3–5 min—have been demonstrated to maximize yields of RA and total phenolics while preserving their antioxidant activities (Jovanović et al. 2022; Ferrentino et al. 2020). Additionally, MAE aligns with green chemistry principles by reducing solvent usage and energy consumption, thus offering sustainability advantages. Due to its effectiveness, reduced extraction times, and environmental friendliness, MAE is becoming increasingly popular for producing high‐purity bioactive extracts in pharmaceutical, nutraceutical, and cosmetic applications (Chaturvedi 2018; Cikoš et al. 2018). MAE is based on the principle of acceleration of solvent penetration and compound diffusions by microwave power. Research has demonstrated that MAE with hydro solvents creates high levels of phenolics with powerful antioxidant potential under optimized conditions (e.g., 371.7 W microwave power, 25%–40% ethanol, and 3–5 min) and minimizes energy and solvent consumption (Caleja, Barros, et al. 2017). MAE extracts have also shown better antioxidant power than those obtained from maceration and ultrasound processes (Jovanović et al. 2022), which supports their growing utilization in the pharmaceutical and nutraceutical area.

### 5.6 Matrix Solid Phase Dispersion

**FIGURE 7:**
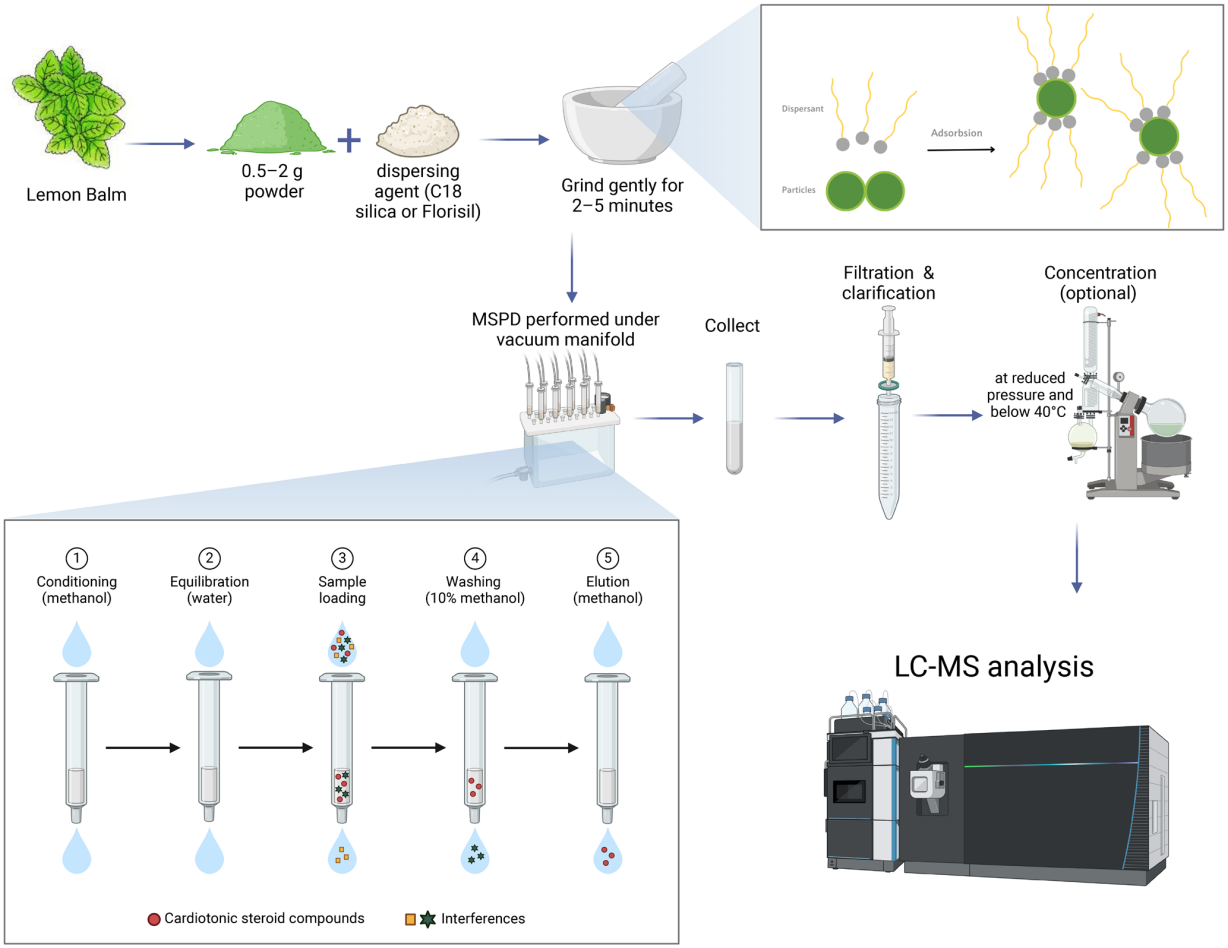
MSPD extraction of *M. officinalis* phenolic compounds schematic. A diagram shows sample preparation, solid‐phase dispersant homogenization, vacuum‐assisted MSPD extraction, filtering, concentration, and LC–MS analysis. Created in BioRender. Awlqadr (2025g) https://BioRder.com/ib0yipn.

Matrix solid‐phase dispersion (MSPD), shown in Figure 7, is a highly efficient and selective extraction method for isolating bioactive compounds, such as phenolic acids (e.g., rosmarinic and caffeic acids), from *M. officinalis* . MSPD involves homogenizing plant material with a solid‐phase dispersant, typically C18 silica or Florisil, facilitating simultaneous extraction and preliminary purification in a single step. Optimal MSPD conditions include gentle blending of plant powder and dispersant, use of ethanol‐water mixtures as eluents, and controlled vacuum‐assisted elution, which achieves high recovery rates, often exceeding 90%. Compared to conventional extraction techniques, MSPD significantly reduces solvent consumption and simplifies the extraction procedure, making it environmentally friendly and cost‐effective. Furthermore, MSPD efficiently produces extracts suitable for subsequent analytical techniques, such as LC–MS, enhancing both the purity and bioactivity of target phenolic compounds (Žiaková et al. 2003). Due to these advantages, MSPD is highly valued for nutraceutical, pharmaceutical, and analytical applications.

Table 6 compares the results of the five advanced extraction techniques (UAE, MAE, PLE, SFE, and MSPD) to classical techniques (maceration, Soxhlet extraction, and hydrodistillation). It emphasizes a side‐by‐side comparison of efficiency, time, solvent consumption, and types of bioactive materials released from *M. officinalis* . In general, newer methods are more efficient, have higher yields, and are better at preserving bioactive compounds, sensitive compounds such as RA and EOs, in particular.

**TABLE 6:** Comparison of new extraction methods to traditional methods (Žlabur et al. 2016; Sik et al. 2020; Miron et al. 2013; Rozzi et al. 2002; Žiaková et al. 2003; Elyemni et al. 2019).

Extraction method	Description	Advantages over traditional methods	Bioactives extracted	Time efficiency	Solvent use
Ultrasound‐assisted extraction (UAE)	Uses high‐frequency sound waves to disrupt plant cells and enhance solvent penetration, reducing extraction time	Faster extraction, higher yield, better preservation of bioactives	Rosmarinic acid, flavonoids, terpenoids, essential oils	30 min to 2 h	Moderate
Microwave‐assisted extraction (MAE)	Uses microwave radiation to rapidly heat solvents and plant material, improving compound extraction efficiency	Faster extraction, preservation of heat‐sensitive compounds like flavonoids	Rosmarinic acid, caffeic acid, essential oils	3–5 min	Low
Pressurized liquid extraction (PLE)	Involves elevated pressure and temperature to improve solvent solubility and extraction efficiency	Higher efficiency, less solvent use, and faster extraction time	Flavonoids, phenolic acids, essential oils	15 min to 1 h	Low
Supercritical fluid extraction (SFE)	Uses supercritical CO_2_ to extract bioactives without high temperatures, reducing thermal degradation	Higher purity of volatile oils and phenolic compounds, less solvent use	Citral, citronellal, flavonoids, phenolic acids	20–60 min	Very low
Matrix solid‐phase dispersion (MSPD)	Involves homogenizing plant material with a solid‐phase dispersant, reducing solvent use and improving purity	Cleaner extracts with better yields of phenolic compounds and lower solvent consumption	Flavonoids, rosmarinic acid, phenolic acids	30 min to 2 h	Very low
Traditional methods	Methods like maceration, Soxhlet, and hydrodistillation, which are slower and use more solvents	Slower extraction, higher solvent use, and higher energy consumption	Rosmarinic acid, flavonoids, phenolic compounds, essential oils	Hours to days	High

### 5.7 Critical Analysis of Extraction Techniques

The selection of an extraction method is of utmost importance to the recovery and bioactivity of the phytochemicals extracted from *M. officinalis* . Traditional techniques such as maceration and steam distillation have been applied, of course, but with some noticeable drawbacks concerning yield and maintaining hydrolyzable compounds such as RA (Kamil Hussain et al. 2019). The majority of these methods require high temperatures that can lead to the degradation of heat‐sensitive compounds and, as a result, diminish the overall biological activity of the extract (Alimoradi et al. 2023). However, SFE, UAE, MAE, and other modern methods provide many advantages, including more effectiveness, selectivity, and bioactivity preservation. For example, SFE has been shown to have high selectivity in the extraction of EOs and phenolic compounds at high purity, and without the need for solvents, this method is an environmentally friendly option. Zaid et al. (2020) used SFE with supercritical CO_2_ to obtain volatile compounds, including geraniol, limonene, and eugenol, from *M. officinalis* , obtaining an extract yield of 1.43% using pressure and temperature at optimum levels. This study highlights SFE as a useful tool to recover selectively the valuable EOs, supporting our stand on the efficiency of extracting some selected bioactive compounds (Zaid et al. 2020). Nevertheless, SFE is expensive and needs specialized equipment, making it inapplicable for industrial application.

UAE and MAE, however, present attractive alternatives to overcome them, improving the extraction efficiency and minimizing the extraction time. The use of these methods to dry *M. officinalis* extracts has been demonstrated to effectively maintain the bioactivity of the red box ( *Eucalyptus polyanthemos* ) leaf fence extracts, especially the antioxidant activity and EO content (Mirghafourvand et al. 2016). Although UAE is key mainly in phenol compound isolation, such as RA, MAE has the added value of greatly shortening the extraction time, thereby increasing productivity on the whole. But an optimization of the extraction parameters (temperature, power, and solvent volume) is required by these methods. For instance, the application of very high microwave power may disrupt labile compounds, and if the solvent ratio is incorrect, the extraction will be incomplete (Qadir et al. 2025). Therefore, it is essential to overview the advantages and disadvantages of these approaches to establish their suitability for various therapeutic applications.

## 6 Pharmacological Applications of *M. officinalis*

**FIGURE 8:**
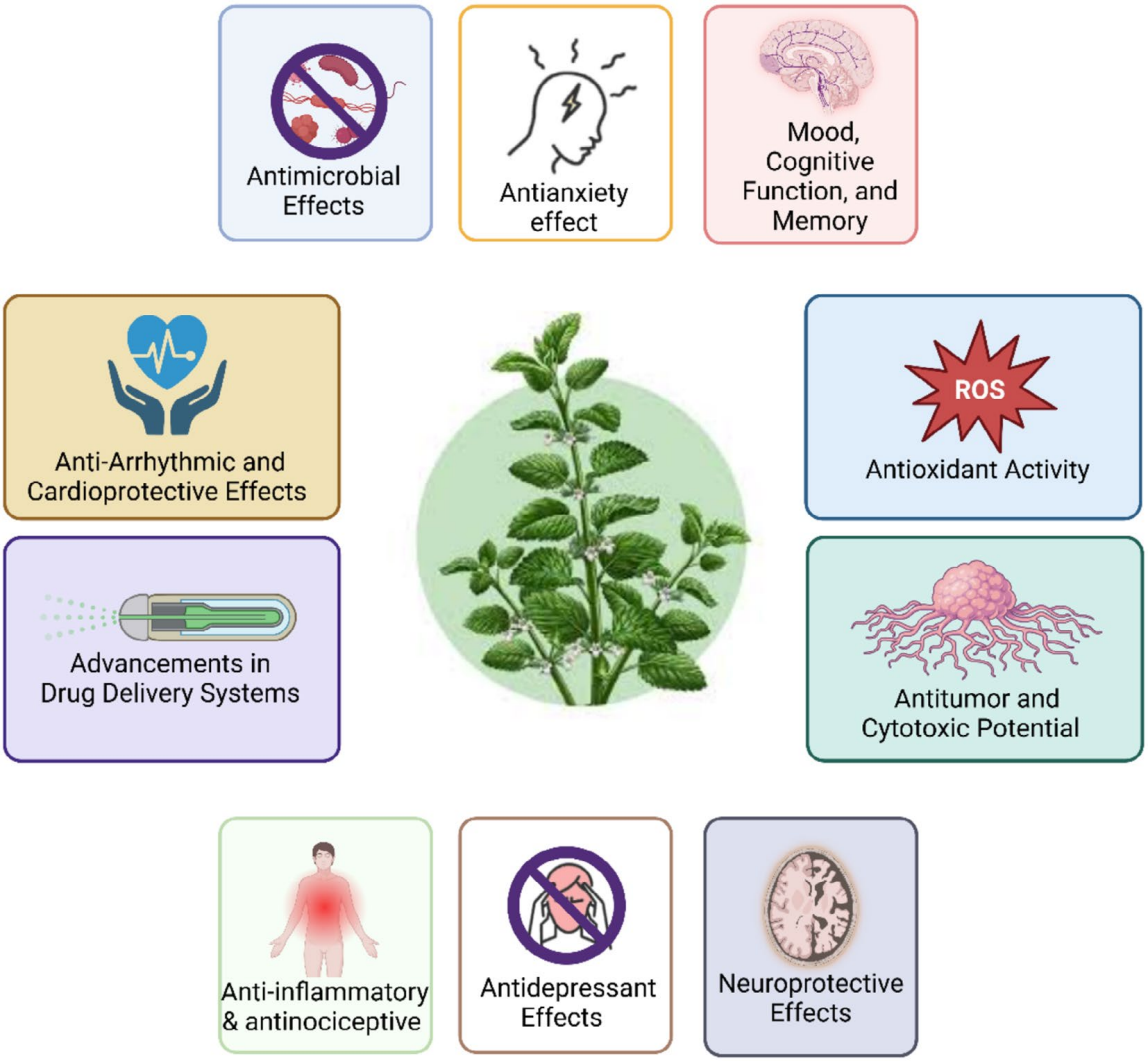
Summary of *M. officinalis* drug applications. The figure shows antioxidant, antimicrobial, anxiolytic, cognitive‐enhancing, antidepressant, neuroprotective, anti‐inflammatory, anticancer, cardioprotective, and the newest drug delivery system effects. Created in BioRender. Awlqadr (2025g) https://BioRender.com/qdfxlhl.

Due to its bioactive profile of several components, *M. officinalis* has many pharmacological activities, as shown in Figure 8. It is efficient against bacteria and fungus and has been incorporated into pharmaceuticals (Khojasteh et al. 2020). In addition to its traditional use in brain problems, *M. officinalis* also improves memory and reduces sadness and anxiety (Ghazizadeh, Sadigh‐Eteghad, et al. 2021). Recent studies show *M. officinalis's* anti‐inflammatory, antinociceptive, cardioprotective, neuroprotective, and anticancer properties (Świąder et al. 2019). This shows that *M. officinalis* is the most widely used plant for its anti‐inflammatory, antinociceptive, cardioprotective, neuroprotective, and anticancer effects. Recently developed medication delivery methods have reduced bioavailable dose loss and boosted *M. officinalis* ‐derived phytochemical efficacy. *M. officinalis's* extensive pharmacological activities make it essential in medicine, stable components, and cosmetics (Ghazizadeh, Sadigh‐Eteghad, et al. 2021; Świąder et al. 2019; Kamdem et al. 2013).

### 6.1 Neuroprotective Effects

The therapeutic use of *M. officinalis* in treating various central nervous system (CNS) disorders is well‐supported by in vitro and in vivo studies demonstrating its neuroprotective properties. Experimental results have confirmed the fact that the use of the methanol extracts of *M. officinalis* in treating the PC12 cells defends against H_2_O_2_‐induced cytotoxicity, as proved by the MTT and LDH assays—methods that are usually used for viability and toxicity assessment in cultured cells under the conditions of oxidative stress (Bayat et al. 2012; Sepand et al. 2013). Both aqueous and methanol extracts of *M. officinalis* remarkably lowered intracellular ROS formation, which also proved to help its neuroprotective potential (Sepand et al. 2013; Geun Kim and Sook Oh 2012). PC12 cell pretreatment with the acidic part of *M. officinalis* ethanol extracts, which have a high content of polyphenols, flavonoids, and terpenoids, has been found to mitigate the Aβ‐induced oxidative damage and cell death. These actions are primarily due to the plant's strong antioxidant activity (Sepand et al. 2013; Shahidi et al. 2019; Soodi et al. 2016). Besides, the run‐in of [3H]‐(N)‐nicotine from acetylcholine receptors in human brain cell membranes by *M. officinalis* extracts illustrates a likelihood of interaction between nicotinic receptors. The ethanol extract manifested an IC50 for nicotine displacement of less than 100 μg/mL, a factor that confirms its efficiency (Kennedy et al. 2003). Nicotine was claimed to have neuroprotection effects against the oxidative stress caused by Aβ and apoptosis, and mostly, it is the nicotinic receptors that are the guilty ones for the same (Kihara et al. 1997; Liu and Zhao 2004). Besides that, the aqueous extract of *M. officinalis* showcased its protective qualities against MDMA‐induced apoptosis in primary hippocampal neurons, the reason being that it can act as a free‐radical scavenger and inhibit monoamine oxidase (MAO) (Hassanzadeh et al. 2011). RA, which is a major phenolic compound in *M. officinalis* , has already been proven to be a real help for human primary neuronal cells when ciguatoxin threatened them by protecting their structural integrity (Taram et al. 2018). The neuroprotective properties of the plant are also further increased by its main phenolic components, represented by quercetin, gallic acid, caffeic acid, chlorogenic acid, and rutin, which as a whole show strong antioxidant and radical scavenging activities. Furthermore, the ability of *M. officinalis* EO to protect against the hypoxia‐caused death of neurons has been recorded in some research. EO remarkably cuts caspase‐3 activity and the number of TUNEL‐positive cells and, thus, diminishes the apoptotic rate. The animal models of ischemia received the treatment of 100 mg/kg of *M. officinalis* EO; this resulted in a fall in malondialdehyde (MDA) levels and elevation in Trolox equivalent antioxidant capacity (TEAC) in the hippocampus (Bayat et al. 2012; Mahita et al. 2014). However, it is essential to be careful because the EO showed a neurotoxic effect in the primary cell cultures at the concentration of 0.1 mg/mL (Mahita et al. 2014; Lima et al. 2004). The mechanism of *M. officinalis* EO protecting the cells might be through the control of the hypoxia‐inducible factor‐1α (HIF‐1α), which influences oxidative stress and apoptosis (Bayat et al. 2012; Queiroz et al. 2014). The primary oxygenated monoterpenes are potent citral isomers and citronellal, major components of the EO. These are the monoterpenes that have been detected as responsible for impacting the antioxidant activity of the plant, counted on the highest extent of their concentration (Ruberto and Baratta 2000; Mimica‐Dukic et al. 2004; Widelska et al. 2018; Ling et al. 2022). Conclusively, *M. officinalis* is neuroprotective due to the antioxidant activity, modulation of nicotinic receptors, and the inhibition of oxidative stress and apoptosis. Thus, these findings have shown that the traditional use of *M. officinalis* to treat neurodegenerative diseases like dementia, epilepsy, stroke, and paralysis cannot be dismissed.

### 6.2 Impact of *M. officinalis* on Mood, Cognitive Function, and Memory

*M. officinalis* was traditionally used in healing different diseases that affect the brain, like dementia and amnesia, which are often the symptoms of Alzheimer's disease (AD) (Ballard et al. 2009). In addition to that, it is the utmost wisdom of doctors to treat diseases of mood, such as with psychosis medications. The principled power of *M. officinalis* is essentially joined with its acetylcholinesterase (AChE) inhibitory activity. This will raise the acetylcholine levels of the synapses in the brain and reduce the cognitive symptoms of AD. This also will help with learning deficits, and it will relieve the symptoms of psychiatric diseases like schizophrenia (Shakeri et al. 2016; Ballard et al. 2009). Abo‐Zaid et al. (2023) reported the neuroprotective role of MEE in the brains of PTU‐induced hypothyroidism and/or IR‐treated rats. Hypothyroidism and/or IR irradiation induced a decrease in serum T3 and T4 levels, increases in MDA and NO as oxidative stress markers, and over‐expression of genes involved in endoplasmic reticulum stress (PERK, ATF6, ERAD, and CHOP) and apoptosis (Bax, BCl2, and caspase‐12). MEE also decreased oxidative stress and endoplasmic reticulum stress, inhibited the gene expression of pro‐apoptotic proteins, and down‐regulated genes associated with neurodegeneration, including MAPT and APP. Histological observation was in agreement with the better brain structure of MEE‐treated rats. Taken together, the results of this study indicated that MEE may alleviate hypothyroidism‐induced brain damage by suppressing oxidative stress, ER stress, and apoptosis (Abo‐Zaid et al. 2023). Ethanol extracts of *M. officinalis* exhibit AChE inhibition in a time‐ and dose‐dependent manner with significant potency (Dastmalchi et al. 2009). Fractionation of these extracts has identified RA and its derivatives as key contributors to AChE inhibitory effects, while flavonoid‐rich fractions of hydro‐alcoholic extracts further enhance this activity (Dastmalchi et al. 2009; Vladimir‐Knežević et al. 2014). Notably, the plant's EO also inhibits AChE in a dose‐dependent manner, with citral—a major component of the EO—and other monoterpenes contributing to this activity (Sareedenchai 2019; Owokotomo et al. 2015). However, aqueous and methanol extracts have shown little AChE inhibitory activity, potentially due to antagonistic interactions among phytochemicals (Kennedy et al. 2003; Gholamhoseinian et al. 2009). Additional mechanisms, such as inhibition of matrix metalloproteinase‐2 (MMP‐2)—an enzyme implicated in AD—by gallic acid, have been reported, further underscoring the multifaceted neuroprotective properties of *M. officinalis* (Shabani et al. 2020; Mahboubi 2019). Beyond its cholinergic effects, *M. officinalis* impacts the GABAergic system. EO components such as trans‐ocimene bind to GABAA receptors, conferring anti‐agitation properties (Abuhamdah et al. 2008; Huang et al. 2008). These interactions enhance its potential for managing agitation and cognitive dysfunction in AD and other disorders. In vivo, ethanol extracts improve learning and memory in rats, reversing scopolamine‐induced deficits in a manner comparable to cholinesterase inhibitors. However, higher doses (> 200 mg/kg) have not demonstrated enhanced efficacy, possibly due to receptor overstimulation or desensitization (Soodi et al. 2014). Flavonoids such as luteolin and ursolic acid further contribute by mitigating β‐amyloid‐induced memory impairment and reducing oxidative stress (Ullah and Hassan 2022; Hossain et al. 2009). Clinical trials support the benefits of *M. officinalis* in AD and cognitive impairment. For instance, a four‐week, double‐blind, placebo‐controlled study revealed significant reductions in agitation and improved quality of life among dementia patients receiving EO aromatherapy (Mahboubi 2019; Ballard et al. 2002). However, conflicting results have been reported; a 12‐week trial found no significant differences in agitation reduction between *M. officinalis* EO, donepezil, and placebo groups, possibly due to the therapeutic influence of touch and social interaction provided in all groups (Ballard et al. 2002; Burns et al. 2011). Another study on healthy individuals demonstrated that acute administration of *M. officinalis* ethanol extract improved mood and cognitive performance, likely due to its receptor‐binding properties (Kennedy et al. 2003; Kennedy and Scholey 2006). Similarly, a 16‐week trial on mild‐to‐moderate AD patients showed reduced agitation and improved cognition with a hydro‐alcoholic extract standardized to citral content (Akhondzadeh et al. 2003). These findings suggest that the memory‐enhancing and cognitive benefits of *M. officinalis* are primarily mediated through AChE inhibition, modulation of nicotinic, muscarinic, and GABAA receptors, and its antioxidative properties. These outcomes validate the classic method of doing this to people and emphasize the drug's enticement as a supplementary therapy for neurodegenerative diseases.

### 6.3 Anti‐Anxiety Effect

*M. officinalis*, also well known as lemon balm, is a medicinal plant that dates back to ancient times as a calming and soothing drug. Recent research has confirmed the soothing effects of *M. officinalis* , making it a promising new solution for addressing anxiety issues (Ghazizadeh, Sadigh‐Eteghad, et al. 2021; Mathews et al. 2024). The use of *M. officinalis* and the main compound, RA, shows that the methanol extracts have GABA transaminase (GABA‐T) inhibitory activity, which means that they can help increase the GABA concentration in the brain, which is a neurotransmitter responsible for stopping anxiety (Awad et al. 2009). In the same way, in vivo studies have proved that the plant's hydro‐alcoholic and ethanolic extracts, taken by mouth, have calming effects brought on by the way the plant functions (Cases et al. 2011). Furthermore, studies in mice have revealed that aqueous extracts of *M. officinalis* can reduce plasma levels of corticosterone, a key stress hormone linked to physiological stress responses (Feliú‐Hemmelmann et al. 2013). Clinical trials support the relevance of these findings to human anxiety management. In a randomized, double‐blind, placebo‐controlled crossover study, 18 healthy volunteers underwent laboratory‐induced stress via the Defined Intensity Stressor Simulation (DISS) battery. After receiving single doses of 300 and 600 mg of standardized methanolic *M. officinalis* extract, participants reported increased calmness and reduced alertness, particularly with the 600 mg dose (Kennedy et al. 2004). Additionally, a prospective open‐label pilot study assessed the effects of Cyracos, a patented standardized extract of *M. officinalis* leaves containing over 7% RA and 15% hydroxycinnamic acid derivatives, in volunteers with mild‐to‐moderate anxiety and sleep disturbances. According to the findings, those on the 15‐day program at 600 mg a day saw an 18% reduction in anxiety, a 15% improvement in anxiety symptoms, and a 42% decrease in insomnia (Cases et al. 2011). It shows that *M. officinalis* is potentially an effective natural treatment for anxiety and to relieve nervousness. Nevertheless, more controlled trials are necessary to decipher additional mechanisms involved in its anxiolytic effects and to understand the impact of other active compounds in the plant. Thus, these results imply that the plant is traditionally used and can pave the way for a substantial implication in drug administration.

### 6.4 Antitumor and Cytotoxic Potential of *M. officinalis*

*M. officinalis* has shown significant potential in cancer treatment through its cytotoxic, antiproliferative, and antimutagenic properties. The EO and its major component, citral, have demonstrated potent effects in vitro by inducing apoptosis in glioblastoma multiforme (GBM) cell lines, particularly those expressing active multidrug resistance protein 1 (MRP1). Citral's action includes generating reactive oxygen species (ROS) and inhibiting MRP1 expression, leading to reduced viability in various human tumor cell lines and a mouse melanoma cell line (Queiroz et al. 2014; De Sousa et al. 2004). The hydro‐alcoholic extract of *M. officinalis* has exhibited antiproliferative effects on colon carcinoma cells by inducing ROS‐mediated apoptosis. However, with IC50 values above 100 μg/mL, its therapeutic potential in clinical applications may be limited (Weidner et al. 2015; Magalhães et al. 2018). Decoctions containing RA and lithospermic acid A have shown growth‐inhibitory activity against diverse human tumor cell lines, further highlighting the role of phenolic compounds in cytotoxic mechanisms (Carocho et al. 2015). In addition, dichloromethane and n‐hexane fractions of *M. officinalis* extract displayed dose‐dependent cytotoxic effects against leukemia cell lines (K562 and Jurkat). The dichloromethane fraction induced apoptosis through upregulation of pro‐apoptotic genes, such as Fas and Bax, and an increased Bax/Bcl‐2 ratio, implicating activation of both extrinsic and intrinsic apoptotic pathways. In contrast, the n‐hexane fraction inhibited cell growth without significantly affecting apoptosis‐related gene expression, suggesting alternate mechanisms of action (Ebrahimnezhad Darzi and Amirghofran 2013). In vivo studies further underscore the antitumor potential of *M. officinalis* . An ethanol extract demonstrated antigenotoxic and antimutagenic effects in mice by reducing genotoxic damage induced by methyl methane sulfonate (MMS), although the effective dose (500 mg/kg) and route of administration (intraperitoneal) are impractical for human translation (Saraydin et al. 2012; de Carvalho et al. 2011). The phenolic compounds, particularly RA, are thought to mediate these effects, with RA significantly reducing chromosomal damage caused by doxorubicin and ethanol in both mice and V79 cells (de Carvalho et al. 2011; Furtado et al. 2010). A study by Kuo et al. (2020) on chemopreventive effects of the components of herbal tea against colorectal cancer (CRC) found that of the nine examined constituents, an *M. officinalis* hot water extract exhibited the most potent anticancer effect against CRC cells. *M. officinalis* suppressed cell growth, induced G2/M cell cycle arrest and caspase‐dependent apoptosis, and induced an inhibition of cell migration through modulating EMT. Mass spectrometry found 67 compounds in the extract, including phenolic compounds such as lignans, phenylpropanoids, and polyketides, that are associated with their antioxidant and anticancer activities. Our results indicate that drinking herbal tea, especially *M. officinalis* , could be beneficial for the prevention and treatment of CRC (Kuo et al. 2020). In addition, this research shows that gallic acid, like a mutation inhibitor, reduces the mutation frequency of DNA repair enzymes and gene expression (Ferk et al. 2011). The experimental data possess the effect of *M. officinalis* extracts, which is selective cytotoxicity. In the same manner, the experiments have not measured the damage to normal cells. This means there is a need for further study to ensure the therapy is safe and potent enough for clinical applications. Moreover, the quantities given for in vivo availability indicate the need for appropriately formulated and synergistically acting compounds to enhance their bioavailability as well as efficacy. These results confirm that *M. officinalis* may be a helpful plant in cancer therapy and suggest that discovering those parts that prevent any further obstacle will be very important to achieve clinical effects (Jahanban‐Esfahlan et al. 2015).

### 6.5 The Role of *M. officinalis* Antioxidant in Disease Prevention

Oxidative stress is a condition that occurs when the production of reactive oxygen species (ROS) is greater than the body's antioxidant defense mechanisms and is thus the main reason for the development of many diseases such as neurodegenerative disorders, cardiovascular diseases, diabetes, and cancers (Birben et al. 2012). A researcher can talk about the harmful effects of ROS like superoxide radicals, which are not radicals; water and hydrogen peroxide, which are the antioxidants that the body synthesizes through reduced glutathione (GSH) and superoxide dismutase (SOD); and when they attack endogenous antioxidants, thus allowing oxidative damage to occur (Slemmer et al. 2008). Some different in vitro and in vivo studies have shown the antioxidant potential of EOs, as well as extracts of *M. officinalis* , indicating the possibility of its use for therapy (Rădulescu et al. 2021). The aqueous ethanol extract of *M. officinalis* has been extensively studied for its antioxidant properties using various assays, including iron (III) reduction, iron (II) chelation, DPPH, ABTS, superoxide anion scavenging, and inhibition of β‐carotene‐linoleic acid bleaching. Notably, its antioxidant activity (90.43 ± 1.55 μg/mL) was comparable to quercetin (98.46% ± 0.89%) and butylated hydroxyanisole (BHA) (96.08% ± 1.58%) and superior to gallic acid and caffeic acid (Fan et al. 2008). *M. officinalis* has a high phenolic content, which is why it has antioxidant activities. This is due to RA, quercetin, gallic acid, caffeic acid, and chlorogenic acid (Ibragić et al. 2014). RA, for instance, manifests robust DPPH scavenging activity with an EC50 of 26.03 μg/mL (Erkan et al. 2008). In the same fashion, caffeic acid shows promising antioxidant activity by decreasing the formation of lipid peroxide and scavenging superoxide radicals at very low concentrations (EC50 values below 5 μg/mL). Also, in another study, quercetin, rutin, and other phenolic compounds were active as DPPH and TBARS scavengers (Girsang et al. 2020). The EO from *M. officinalis* is the most appropriate in this way since it has the most active DPPH scavenging ability (IC50 value of 7.58 μg/mL). This activity results from the monoterpene aldehydes (e.g., citral isomers), ketones (e.g., isomenthone, menthone) in small percentages, and mono‐ and sesquiterpene hydrocarbons (Mimica‐Dukic et al. 2004). Apart from the above, in a remarkable study, the infusion of *M. officinalis* reduced the oxidative stress markers in the radiology staff that had been exposed to the high level of ionizing radiation. Medical treatment remarkably improved the SOD, catalase, and GSH peroxidase levels, and at the same time, reduced the hepatic DNA damage, lipid peroxidation, and myeloperoxidase activity (Zeraatpishe et al. 2011). These results reveal that *M. officinalis* antioxidant functions against oxidative stress of the nervous and heart systems that lead to diseases, including AD and CAD, can be associated with its spectacular ability to be a strong scavenger of free radicals, to be a suitable inhibitor of lipid peroxidation, and to be an endogenous antioxidant enzyme activator (Miraj et al. 2017).

### 6.6 Antimicrobial Effects

*M. officinalis* has strong and effective antimicrobial elements, showing a variety of antiviral, antibacterial, and antifungal actions. The EO of *M. officinalis* was found to have good in vitro activity against Gram‐negative bacteria, including *Pseudomonas aeruginosa, Salmonella enteritidis, Salmonella typhi, Escherichia coli*, and *Shigella strains*, but the highest activity was detected against *E. coli* and multi‐resistant strains of *Shigella sonnei* (Mimica‐Dukic et al. 2004). Antibacterial effects of the EO are also applicable to food‐borne pathogens and spoilage bacteria with minimum inhibitory concentration (MIC) values ranging from 72.0 to 1000.3 μg/mL, which are the same as those displayed by *Rosmarinus officinalis* but are less potent than ciprofloxacin (MIC: 2.5–62.2 μg/mL; Hussain et al. 2011). The EO's antimicrobial activity is largely attributed to its citral (geranial and neral) and citronellal content, which disrupt microbial membranes and impair cellular processes (Mimica‐Dukic et al. 2004). Extracts of *M. officinalis* (e.g., petroleum ether, chloroform, ethyl acetate, and n‐butanol) demonstrate antibacterial activity against Gram‐positive strains such as *S. lutea*, *S. aureus* , and *B. cereus* (zone inhibition diameters: 10.7–19.3 mm), with ethyl acetate and n‐butanol extracts being rich in RA, caffeic acid, and p‐coumaric acid (Čanadanović‐Brunet et al. 2008). Decoctions containing RA and lithospermic acid A exhibit significant activity against *P. aeruginosa* , *Salmonella typhimurium*, and *Penicillium funiculosum*, with MIC and minimum bactericidal concentration (MBC) values comparable to or surpassing streptomycin and ampicillin (Carocho et al. 2015). Moreover, sulfated terpenes from hydro‐alcoholic extracts show antifungal activity against *Candida* species ( *C. albicans* , *C. glabrata* , and *C. krusei* ) and *Aspergillus fumigatus* and antibacterial effects against *Mycobacterium intracellulare* , *E. coli* , *P. aeruginosa* , and *S. aureus* , though less potent than ciprofloxacin (MIC > 1.5 μg/mL vs. 0.98 μg/mL for ciprofloxacin; Tantry et al. 2014). Furthermore, the antiviral activity of *M. officinalis* has been reported against herpes simplex virus type 1 (HSV‐1) and influenza A virus, with the EO and extracts interfering with viral replication and promoting host cell resistance (Allahverdiyev et al. 2004). Behzadi et al. (2023) examined the antiviral activity of *M. officinalis* (lemongrass), a plant that has been used traditionally to treat stress, anxiety, appetite, sleep, pain, wounds, and poisonous insect bites. Standing the increase in use of synthetic drugs, herbal drugs are still being preferred by some people due to their fewer side effects, lower cost, and history as traditional medicine. Lemongrass has been demonstrated recently to be active against several viruses such as SARS‐CoV‐2, herpes simplex virus (HSV), and human immunodeficiency virus (HIV) by inhibiting HSV‐1 attachment to the host cells, inhibiting post‐adsorption of HSV‐1 in the replication cycle, and inhibiting the main protease and spike protein of SARS‐CoV‐2. The review indicates that *M. officinalis* is potentially applied in curing relative diseases; however, a great deal of in vitro and in vivo research would be needed to better clarify its mechanism of action. Moreover, more randomized controlled trials have been required to evaluate the effectiveness in people. Considering its benefits and safety profile, it may be used as an adjuvant therapy (Behzadi et al. 2023). Table 7 EO of *M. officinalis* was very effective against bacteria, fungi, and yeast. The oil could inhibit Gram‐positive and Gram‐negative bacterial strains, like *Pseudomonas aeruginosa, Escherichia coli, and Klebsiella pneumoniae*. Moreover, the oil displayed good antifungal properties, mainly with *Mucor ramannianus* and *Fusarium oxysporum* strains, and performed strong inhibitory effects on yeast species such as *Saccharomyces cerevisiae* and *Candida albicans* . The most minor concentration at which inhibition was observed was between 1 and 5 μL/mL, underlining the robust antimicrobial activity of this EO, thus indicating potential pharmaceutical and food preservation applications. These results align with the traditional use of *M. officinalis* in treating infections, wounds, and fevers, proving its efficacy as a natural preservative and a therapeutic agent for microbial and viral infections.

**TABLE 7:** Effectiveness of the *M. officinalis* essential oil against particular bacteria, fungi, and yeasts.

Microorganism	Zone of inhibition (mm)	MIC (μL/mL)
Gram‐positive bacteria		
*Staphylococcus aureus*	18–20	3
*Bacillus subtilis*	17	2
*Listeria monocytogenes*	17	2
Gram‐negative bacteria		
*Pseudomonas aeruginosa*	19–31	2–4
*Escherichia coli*	18.5–20	2–4
*Klebsiella pneumoniae*	14.5–21	3
*Salmonella enterica*	9–14	5
Yeasts		
*Candida alicans*	32.5–36	3
*Saccharomyces cerevisiae*	36–38	2
Fungi		
*Fusarium oxysporum albedinis*	30–38	2
*Fusarium oxysporum lini*	21–34	1
*Mucor ramannianus*	29–39	1

*Note:* The zone of inhibition is shown in mm, and the minimum inhibitory concentrations (MIC) (Abdellatif et al. 2014; Alizadeh Behbahani and Shahidi 2019).

### 6.7 Antidepressant Effects of *M. officinalis*

The traditional use of *M. officinalis* as an exhilarant and enlivening agent has been documented in ancient medical texts, reflecting its long‐standing reputation as a mood enhancer. Modern research provides evidence supporting these traditional claims. In an in vitro study, aqueous and methanol extracts of *M. officinalis* demonstrated mild inhibition of monoamine oxidase (MAO)‐A, an enzyme involved in degrading neurotransmitters like serotonin and norepinephrine. The methanol extract exhibited greater potency, with IC50 values of 19.3 μg/mL compared to 48.2 μg/mL for the aqueous extract (López et al. 2009). However, these concentrations are unlikely to be achievable in clinical settings without highly bioavailable formulations, emphasizing the need for optimized preparations. In behavioral studies, the ethanol extract of *M. officinalis* showed significant antidepressant activity in the forced swimming test (FST), a widely used model for evaluating anti‐depressant effects in animals. This effect was linked to enhanced norepinephrine neurotransmission (Emam and Talebianpour 2009). Furthermore, RA, one of the primary active components of *M. officinalis* , reduced immobility time in the FST in mice, suggesting an antidepressant mechanism distinct from monoamine transport or MAO inhibition (Emam and Talebianpour 2009). The research implied the possibility of RA functioning using other neurobiological pathways, such as flagging the inflammatory markers or the oxidative state. Suchendum has been promising for a long time, but then problems emerged because the specific mechanisms were not understood enough for the antidepressant mechanism of *M. officinalis* . It is supposed that producers of AO inhibitors should examine the sluggishness of polar components of the plant, the ability of coumestrols to induce the MAO‐A inhibitory effect, and neurobiological targets (Kera et al. 2024). Although animal studies have registered the antidepressant activity of *M. officinalis* in doses posteriorly up to 25–300 mg/kg, acute IC50 or EC50 values, on the other hand, are not being presented, which prevents them from being taken advantage of. It is crucial to state correctly that although the development of allometric scaling theories in this field allows us to convert the above doses that are to be used theoretically for humanity and also partly consonant with the theory that this scientific name and the traditional usage of this plant are for mood enhancement, though being pensive, more trials ought to be conducted to make its main mechanisms more transparent, to optimize its clinical application, and to improve its therapeutic effectiveness (Emam and Talebianpour 2009; Table 8). *M. officinalis* leaves grown organically showed significantly higher total phenolic and flavonoid content, enhanced antioxidant capacities (ABTS, DPPH, FRAP), and elevated levels of ascorbic acid compared to conventional practices. These findings emphasize the positive impact of organic cultivation in enhancing the antioxidant profile and nutritional quality of *M. officinalis* . This would include a more rigorous exploration of dose–response relationships and the pharmacokinetics of its active constituents.

**TABLE 8:** Total phenolics, flavonoids, and antioxidant activity of *M. officinalis* leaves during the first harvest (Chrysargyris et al. 2022; Silva et al. 2023).

Parameters	Concentration range (Mean ± SD)
Total phenols	225.53–350.94 ± 25.61
Total flavonoids	14.21–26.75 ± 1.56
ABTS (mg Trolox eq./g FW)	27.95–36.58 ± 1.54
DPPH (mg Trolox eq./g FW)	48.21–74.48 ± 3.41
FRAP (mg Trolox eq./g FW)	88.36–135.34 ± 13.95
Ascorbic acid (mg/g FW)	33.03–66.05 ± 2.60
TBARS	125–206 ± 2.66
OxHLIA	13.5–61.4 ± 0.43

*Note:* Values represent mean ± SD (*n* = 3).

Abbreviations: FW, fresh weight; Trolox eq., Trolox equivalent.

### 6.8 Cardiovascular Impacts of *M. officinalis* : Anti‐Arrhythmic and Cardioprotective Effects

*M. officinalis* is widely regarded as a heart tonic in traditional medicine and has demonstrated notable cardioprotective and anti‐arrhythmic properties. Pharmacological studies highlight its potential to regulate heart rhythm, reduce palpitations, and mitigate myocardial injury. For instance, an ethanol extract of *M. officinalis* significantly decreased the incidence of ventricular premature beats (VPB), ventricular fibrillation (VF), and ventricular tachycardia (VT) in rats subjected to CaCl2‐induced arrhythmias, with these effects attributed to β‐adrenergic antagonistic activity (Akhondali et al. 2015; Joukar et al. 2014; Sedighi et al. 2019). Further, due to the high content of RA and phenolics in the aqueous extract obtained from the plant, QRS, QTc, JT, and TpTe intervals overlapped on the ECGs and in vivo, probably due to the fact that the plant extract induced proper ion channel activities in the rat heart and reduced (Joukar and Asadipour 2015). This relevance elucidates the clinical applicability, although well‐designed human trials are necessary to investigate such mechanisms. In the past, the bradycardic effects of *M. officinalis* were detected in the hearts of isolated rats, which were later revealed in humans in a sense where there were no substantial heart rate decrements. However, it showed a reduction in the frequency of benign palpitations, and it has been established to be a very safe method for patients with benign arrhythmias (Gazola et al. 2004; Aliabadi and Zendehboodi 2021). Extra doses of the fluid also offered a little protection against rats' ventricular arrhythmias during the reperfusion phase, probably by blockage of muscarinic receptors (Joukar et al. 2014). This anti‐arrhythmic effect may occur through interfering with β‐adrenergic regulation and parasympathetic modulation of beta and sodium and potassium modulation via conductivity. Additionally, along with reducing blood pressure, *M. officinalis* was reported to demonstrate vasorelaxant effects on the isolated rat aorta by activating endothelial nitric oxide and possibly prostacyclin and EDHF (Ersoy et al. 2008). These structures make the herb capable of lowering high vascular resistance and thus increasing blood flow into the heart. In addition, the animal's treatment with a dose of 50 mg/kg of the plant's aqueous extract made it more resistant to isoproterenol‐induced myocardial injury by reducing malondialdehyde (MDA) levels and improving the oxidative balance. Consequently, higher doses (200 mg/kg) were more harmful because possibly they increased heart contractility and oxygen demand (Joukar and Asadipour 2015). Strikingly, RA, the primary compound in *M. officinalis* , has been demonstrated to prevent myocardial fibrosis and to restore heart function in insulin‐resistant animals. RA achieves these effects by reducing oxidative stress, angiotensin II levels, collagen deposition, and fibrogenic markers such as transforming growth factor‐β1 (TGF‐β1) and matrix metalloproteinases (MMPs). Furthermore, RA enhances the expression of tissue inhibitors of metalloproteinases (TIMPs), thereby counteracting fibrotic processes (Chacko et al. 2012; Boo 2024). As fibrosis contributes to heart failure and arrhythmias, RA's antifibrotic effects may underlie some of the cardioprotective benefits attributed to *M. officinalis* . These findings align with traditional uses of *M. officinalis* for the treatment of heart palpitations, anxiety‐related cardiac symptoms, and general heart health. The plant's antioxidant, hypolipidemic, and anti‐anxiety properties may further enhance its therapeutic potential for cardiovascular diseases. Nonetheless, future studies should focus on elucidating the dose‐dependent effects, long‐term safety, and clinical efficacy of *M. officinalis* in human populations to validate its cardioprotective applications fully.

### 6.9 Anti‐Inflammatory and Antinociceptive Properties of *M. officinalis*

*M. officinalis* has been traditionally utilized for managing inflammatory conditions such as asthma, joint inflammation, and pain, underscoring its potential as an anti‐inflammatory and analgesic agent. Contemporary pharmacological studies validate these traditional claims, revealing the plant's significant anti‐inflammatory and antinociceptive activities. Studies demonstrate that pretreatment with aqueous extracts of *M. officinalis* effectively reduces inflammation‐induced paw edema in rats, likely through the modulation of inflammatory mediators (Birdane et al. 2007; Bounihi et al. 2013; Draginic et al. 2022). Ethanol extracts also exhibit anti‐inflammatory properties, as evidenced by a dose‐dependent reduction in carrageenan‐induced paw edema and trauma‐induced inflammation in rats. While tested doses (200–400 mg/kg) may be challenging for direct human application, no adverse effects were reported at doses as high as 2000 mg/kg, indicating a favorable safety profile (Bounihi et al. 2013). The anti‐inflammatory activity of *M. officinalis* is closely linked to its bioactive components. Citral, a major constituent of the plant's EO, inhibits pro‐inflammatory cytokines such as TNF‐α, IL‐6, and IL‐1β in lipopolysaccharide (LPS)‐stimulated macrophages, suggesting its role in suppressing the inflammatory response (Bounihi et al. 2013). Additionally, RA, another key phytochemical, exerts anti‐inflammatory effects by inhibiting cyclooxygenase (COX) enzymes, reducing prostaglandin synthesis, and scavenging free radicals, thereby mitigating oxidative stress, a key driver of inflammation (Guginski et al. 2009). *M. officinalis* also demonstrates pronounced antinociceptive properties. Ethanol extracts exhibit dose‐dependent antinociceptive effects in chemical nociception models in mice, mediated through inhibition of the L‐arginine‐nitric oxide pathway and activation of the cholinergic system (Guginski et al. 2009). It is revealed that RA is primarily responsible for these effects, and its effective dose (ED50) of 2.6 mg/kg is clinically relevant compared to the higher doses required for crude extracts (Guginski et al. 2009). For instance, oral administration of *M. officinalis* EO (0.01–0.04 mg/day) in the long term reduces diabetic hyperalgesia in diabetic‐induced animal models, demonstrating its analgesic potential (Hasanein and Riahi 2015). In addition, the study showed the effect of hydro‐alcoholic extract of *M. officinalis L*. against diabetes‐induced learning and memory impairment in rats. It was applied orally at doses of 25, 50, or 100 mg/kg for 2 weeks, following a dose–response study using the highest nontoxic dose. Treated diabetic rats exhibited a significant enhancement of memory performance as evidenced by the performance in Y‐maze and passive avoidance tasks. Moreover, treatment with *M. officinalis* normalized hippocampal expression of brain‐derived neurotrophic factor (BDNF) and nitric oxide synthase (NOS) that were decreased by diabetes. This evidence confirms the known nootropic activity of *M. officinalis* and its possible anti‐diabetic therapeutic effect against memory dysfunction (Naseri et al. 2021). It is thought that the analgesic action is possibly due to the inhibition of neurotransmission in the pain nerve pathways and the release of various inflammatory factors. EOs such as citral have been found to be antinociceptive in EO‐favored and pain‐averted animals. *M. officinalis's* anti‐inflammatory and antinociceptive activities are due to its direct modulation of key inflammatory mediators and signaling pathways. Such midpoints are the reduction of TNF‐α, IL‐6, and IL‐1β by citral, and the reduction of nitric oxide synthesis by RA in light of the plant's multi‐pronged selective pharmacological properties (Bounihi et al. 2013; Guginski et al. 2009). Further complementing RA's anti‐inflammatory and analgesic efficacy is its ability to inhibit both COX enzymes and eliminate free radicals (Guginski et al. 2009). Still, even the findings are exciting at the preclinical level, but applying these effects to human scales needs more work. Besides this, doses like those in animal models may not, however, be practicable in humans unless they are logically formulated for bioavailability and safety. Thus, no toxicity that appears at high doses in preclinical studies in human beings would be glad to tell that *M. officinalis* is a safe candidate for further research. All in all, the findings confirm the traditional use of *M. officinalis* for inflammatory and nociceptive conditions, and its anti‐inflammatory effects are primarily due to compounds like citral and RA, and its pathways include nitric oxide inhibition and cytokine suppression. Hence, it is its therapeutic potential.

### 6.10 Therapeutic Applications of *M. officinalis* for Obstetric and Gynecological Health

*M. officinalis* has attracted increased interest in recent years because of its potential as a therapeutic for obstetric and gynecological conditions. Historically, this herb has been used as a traditional remedy, which has shown several pharmacological activities, and could be a good candidate in controlling the complications that might affect women (Alimoradi et al. 2023). Several studies have investigated its use for pain relief, improved mood, and management of symptoms associated with premenstrual syndrome (PMS), postpartum depression (PPD), and menopausal discomfort. There are multiple randomized controlled trials and systematic reviews that adduce support for the effectiveness of *M. officinalis* in the treatment of primary dysmenorrhea. The results of the study by Mirghafourvand et al. (2016) demonstrated the beneficial effect of *M. officinalis* extract on the severity of menstrual pain in women suffering from primary dysmenorrhea. Also, another double‐blind, randomized trial by Dastjerdi et al. (2019) showed that *M. officinalis* capsules were effective in reducing pain and enhancing the quality of life of women with moderate to‐severe menstrual pain. It has analgesic properties, thought to be due to its prostaglandin‐inhibiting properties (prostaglandins are mediators of inflammation and pain associated with menstruation). The latter results were also corroborated by Alimoradi et al. (2023), who similarly reported the role of *M. officinalis* in softening the severity of menstrual cramps. In addition to period pain, *M. officinalis* is being investigated as a treatment for the psychological symptoms that accompany multiple conditions related to gynecological health, such as PMS and postpartum depression (Dehcheshmeh and Parvin 2016). A study by Heydari et al. (2019) found that Byakuin had significant anxiolytic effects and relieved other psychosomatic complaints in adolescent females with PMS. The effects were also enhanced by those findings of a study by Akbarzadeh et al. (2018) that reported that daily *M. officinalis* capsules reduced mood disturbances and improved mental health in women suffering from PMS. The anxiolytic effect of *M. officinalis* appears to be mediated through the modulation of neurotransmitters including, gamma‐aminobutyric acid (GABA) and serotonin, which are important in emotional control. This pharmacological action implies that lick resin can be used as a potent substitute for women who are looking for alleviation of their psychological symptoms of PMS without the negative side effects typically encountered with pharmacology. Postpartum depression (PPD), another common condition among new mothers, is a type of depression that causes sadness, anxiety, and emotional instability. Mirabi et al. (2017) reported that mood and anxiety symptoms of PPD were mitigated by the use of *M. officinalis* extract. This and other studies, in turn, raise the possibility that the herb's calming effects could help women get a handle on postpartum blues, a common early post‐childbirth affliction. The anxiolytic and weak sedative activities of *M. officinalis* have been linked to its high level of bioactive compounds, such as RA, a polyphenol with well‐established neuroprotective and anti‐inflammatory effects. Regarding menopausal symptoms, the evidence is conflicting with respect to *M. officinalis* efficacy (e.g., as for anxiety and mood swings, but not per se other symptoms including hot flashes and night sweats). An RCT by Darvish‐Mofrad‐Kashani et al. (2018) reported an improvement of sexual function and desire in women with menopausal symptoms following *M. officinalis* , indicating its potential for managing sexual dysfunction in this phase of the female life cycle. However, further high‐quality trials are required to completely estimate its efficacy in the treatment of menopausal symptoms.

*M. officinalis* may also be promising in cases of sexual dysfunction. Afshar et al. (2020) reported that lemon balm markedly increased sexual desire, lubrication, and satisfaction in women with decreased sexual desire. This was believed to be due to the herb's purported ability to enhance mood and overall psychological well‐being and its mild aphrodisiac effects. In the same line, a work by Darvish‐Mofrad‐Kashani et al. (2018) found that an extract of *M. officinalis* contributed to the better sexual performance of women with sexual dysfunction related to psychological or hormonal causes. However, as optimistic as these results may be, it is worth considering that the data on LB effects on gynecological health are not free of some constraints. Several studies are based on small numbers of patients or may have methodological shortcomings that can influence the generalizability of the results. For example, the research of Alimoradi et al. (2023) said in their systematic review that despite *M. officinalis* showing potential against pain and psychological symptoms associated with gynecological conditions, the overall studies' quality was fair because of issues such as selection bias and poor control groups. This emphasizes the necessity for more studies using larger sample sizes, stronger active techniques, and research investigating the ideal dosage, form, and treatment period of *M. officinalis* for gynecological complaints.

## 7 Conclusions

*M. officinalis* is a highly regarded medicinal herb because of its rich composition of bioactive ingredients, such as phenolic acids, flavonoids, volatile oils, and triterpenoids. Utilizing modern extraction methods such as UAE, MAE, PLE, SFE, and MSPD provides significant benefits compared to traditional techniques, leading to improved extraction yields, compound purity, bioactivity, and environmental sustainability. Research has confirmed its powerful properties, including antioxidant, antimicrobial, anti‐inflammatory, neuroprotective, antiviral, anticancer, antidepressant, anxiolytic, cardioprotective, and cognitive‐enhancing effects, highlighting its substantial therapeutic potential. However, there is still a need for further interdisciplinary studies to refine extraction methods, standardize bioactive content, carry out comprehensive clinical trials, and investigate advanced drug delivery systems. This advancement will enable the successful incorporation of *M. officinalis* ‐derived compounds into a variety of nutraceutical, pharmaceutical, and cosmetic applications, establishing it as a crucial element in contemporary healthcare and functional product sectors. In addition, the traditional applications of *M. officinalis* in treating neurological disorders, including dementia, amnesia, and other symptoms associated with Alzheimer's disease, underscore its potential as a natural neuroprotective agent, warranting further scientific exploration and clinical validation.

## 8 Recommendations and Future Directions

Several targeted and evidence‐based actions are recommended to maximize the benefits of *M. officinalis*. As concluded in this review, the most urgent step is to optimize extraction methods for the selective recovery of key bioactive compounds—particularly RA for its strong neuroprotective and anti‐inflammatory effects, citral‐rich eEOs for antimicrobial activity, and ursolic acid for anticancer potential. Our analysis indicates that integrating green technologies, such as UAE combined with MAE, can significantly increase yields, preserve the stability of heat‐sensitive compounds, and reduce solvent and energy consumption, thereby meeting both therapeutic and sustainability goals. The second priority is to standardize bioactive content across extracts through validated quantification protocols, ensuring consistent potency for pharmaceutical and nutraceutical production and improving reproducibility across research studies. Third, while preclinical evidence already supports the use of *M. officinalis* in managing neurodegenerative diseases, anxiety, and mood disorders, well‐designed animal studies and human clinical trials are required to confirm these effects, establish safe and effective dosage ranges, and validate clinical applicability. Finally, in alignment with our conclusion on enhancing therapeutic delivery, novel formulation strategies—such as nanoparticle encapsulation and liposomal carriers—should be developed to improve the bioavailability and targeted delivery of these bioactives, ultimately maximizing their clinical impact.

## Author Contributions

**Farhang Hameed Awlqadr:** conceptualization, visualization, software, writing – review and editing (equal). **Ammar B. Altemimi:** conceptualization, writing – review and editing (equal). **Syamand Ahmed Qadir:** writing – review and editing (equal). **Othman Abdulrahman Mohammed:** writing – original draft (equal). **Mohammed N. Saeed:** writing – original draft (equal). **Mohammad Ali Hesarinejad:** conceptualization, writing – review and editing (equal). **Naoufal Lakhssassi:** writing – original draft (equal), writing – review and editing (equal).

## Conflicts of Interest

The authors declare no conflicts of interest.

## Data Availability

The data that support the findings of this study are available on request from the corresponding author.
